# A systematic review of hand hygiene improvement strategies: a behavioural approach

**DOI:** 10.1186/1748-5908-7-92

**Published:** 2012-09-14

**Authors:** Anita Huis, Theo van Achterberg, Marijn de Bruin, Richard Grol, Lisette Schoonhoven, Marlies Hulscher

**Affiliations:** 1 Scientific Institute for Quality of Healthcare, Radboud University Nijmegen Medical Centre, Nijmegen, The Netherlands; 2 Communication Science, Wageningen University, Wageningen, The Netherlands; 3 Faculty of Health Sciences, University of Southampton, Southampton, UK

**Keywords:** Hospital-acquired infection, Behaviour, Quality improvement, Handwashing, Program evaluation, Review

## Abstract

**Background:**

Many strategies have been designed and evaluated to address the problem of low hand hygiene (HH) compliance. Which of these strategies are most effective and how they work is still unclear. Here we describe frequently used improvement strategies and related determinants of behaviour change that prompt good HH behaviour to provide a better overview of the choice and content of such strategies.

**Methods:**

Systematic searches of experimental and quasi-experimental research on HH improvement strategies were conducted in Medline, Embase, CINAHL, and Cochrane databases from January 2000 to November 2009. First, we extracted the study characteristics using the EPOC Data Collection Checklist, including study objectives, setting, study design, target population, outcome measures, description of the intervention, analysis, and results. Second, we used the Taxonomy of Behavioural Change Techniques to identify targeted determinants.

**Results:**

We reviewed 41 studies. The most frequently addressed determinants were knowledge, awareness, action control, and facilitation of behaviour. Fewer studies addressed social influence, attitude, self-efficacy, and intention. Thirteen studies used a controlled design to measure the effects of HH improvement strategies on HH behaviour. The effectiveness of the strategies varied substantially, but most controlled studies showed positive results. The median effect size of these strategies increased from 17.6 (relative difference) addressing one determinant to 49.5 for the studies that addressed five determinants.

**Conclusions:**

By focussing on determinants of behaviour change, we found hidden and valuable components in HH improvement strategies. Addressing only determinants such as knowledge, awareness, action control, and facilitation is not enough to change HH behaviour. Addressing combinations of different determinants showed better results. This indicates that we should be more creative in the application of alternative improvement activities addressing determinants such as social influence, attitude, self-efficacy, or intention.

## Background

Hospital-acquired infections (HAIs) burden patients, complicate treatment, prolong hospital stay, increase costs and can be life threatening [1]. Recent studies in Europe have shown that HAIs affect 4.6% to 9.3% of the hospitalised patients [2-8]. In Europe, the estimated five million HAIs that occur annually have an assumed attributable mortality of 50,000 to 135,000 at a cost of €13 to €24 billion [9]. In the United States, prevalence rates were estimated at 4.5% for 99,000 cases of excess mortality and an economic burden of US $6.5 billion in 2004 [10,11].

Adequate hand hygiene (HH) among hospital personnel could prevent an estimated 15% to 30% of the HAIs [12,13]. Numerous studies over the last few decades have shown that HH compliance rates are generally less than 50% of all the opportunities [14-16]. Many strategies have been designed and evaluated to address the problem of low compliance, but most of the effects are small to moderate and often short term [12-17]. This stresses the importance of a clear evidence-based strategy to improve HH routines [18,19].

In 2001, Naikoba and Hayward systematically reviewed 21 studies, all aimed at improving the HH of healthcare workers (HCWs) [20]. The authors concluded that multifaceted strategies are generally more effective than single strategies. Moreover, strategies directed at educating and motivating HCWs, such as written educational materials, reminders, and continuous feedback about performance, were found to be more useful than strategies aimed at offering more facilities such as automated sinks or moisturised soaps. Despite the importance of this review, Naikoba and Hayward’s concluded that most of the reviewed studies had multiple design limitations, which made causal inferences about the effects of strategies problematic. Gould *et al.* also recognised methodological weaknesses of HH studies in their systematic review [21]. However, they conducted a Cochrane review with such stringent criteria that only four studies were included, and many possibly relevant non-randomised trials were disregarded. Therefore, the results of their review provide little guidance to policymakers and hospital staff for designing effective programmes to improve HH adherence. Thus, although high methodological quality is important, reviewers should balance this with the urgency of offering guidance/potential solutions to the field. An update of the literature, balancing methodological quality and the need for evidence, seems warranted. In order to identify effective routes to promoting HH and thereby reduce HAIs, it is important to search the content of improvement strategies that is correlated with improved HH behaviour across studies. In implementation research, the most used classification of strategies is captured in the Data Collection Checklist of the Effective Practice Organisation of Care Group (EPOC), which is based on the form of performed improvement activities [22]. A disadvantage of ‘just’ coding improvement activities as the EPOC describes, is that information about the corresponding triggers that prompt HH behaviour is disregarded. Improving HH compliance implies behaviour change; therefore, application of knowledge from the behavioural and social sciences appears valuable [23-25]. An alternative way of classifying strategies is on the basis of their determinants of behaviour change (Table 1). These determinants are derived from behaviour and behaviour-change theories and describe the way or trigger to arrive at behaviour change [26-29]. This behavioural approach might shed new light on the nature of improvement strategies and elucidating how these strategies work. For example, regularly displaying charts of HH performance on group levels or information about nosocomial infection rates can be considered ‘feedback.’ Reviewing the individual HH compliance and promoting a comparison of HH compliance among team members can also be categorised as ‘feedback.’ However, in the first example, the determinant of behaviour change is ‘raising awareness,’ while the determinant in the second example is ‘social influence.’ Both examples thus target different determinants of behaviour change, but both would be categorised as ‘feedback’ in the EPOC classification system.

**Table 1:** Explanation of terms

**Term**	**Explanation**	**Examples**
**Determinants of behaviour change**	The determinants targeted by a systematically developed strategy are those that have been identified for altering behaviours. Theoretically, the application of a chosen behaviour change activity as part of the HH improvement strategy will alter a specific behavioural determinant, which in turn will change behaviours	Knowledge
		Awareness
		Self-efficacy
**Behaviour change technique**	Behaviour change techniques refer to the specific methods used to promote behaviour change	Education
		Feedback
		Guided practice
**Activities**	Activities refer to the operationalisation of behaviour change techniques	Lectures
		Overview of HH compliance rates
		Teaching skills/specific instruction
**Hand hygiene improvement strategy**	A strategy consist of a set of one or more techniques (*e.g.*, education, feedback, goal setting), intended to change specific determinants (*e.g.*, education to increase knowledge, feedback to raise awareness, guided practice to enhance self-efficacy) of HH behaviour	

Theoretically, the application of a chosen behaviour change activity as part of the HH improvement strategy (*e.g.*, a meeting to educate staff on the World Health Organization five moments for HH) will alter a specific behavioural determinant (in this case, their knowledge on the five moments for HH), which in turn will change behaviours (in this case, HH behaviour in line with the five moments for HH) [26]. We hypothesise that a HH improvement strategy targeting more different determinants of behaviour change will be more effective in increasing HH compliance than a HH improvement strategy targeting less different determinants of behaviour change.

The purpose of the present study is to offer sufficient conceptual clarity on the nature of HH improvement strategies by classifying their improvement activities on the basis of their determinants of behaviour change. In addition, we used the controlled studies of our review to explore the effectiveness of targeting different determinants of behaviour change.

## Methods

### Search strategy

First, we selected the 21 studies that Naikoba and Hayward reviewed [20]. Second, we searched the databases of MEDLINE, CINAHL, EMBASE, the Cochrane Central Register of Controlled Trials (CENTRAL), Cochrane Database of Systematic Reviews, Database of Abstract of Reviews of Effects (DARE) from January 2000 up to November 2009, as well as the Current Controlled Trials, ClinicalTrials.gov, National Health Service Centre for Reviews and Dissemination (NHS-CRD): National Health Service Economic Evaluation Database (NHS-EED), and National Health Service Centre for Reviews and Dissemination Health Technology Assessment (NHS-CRD: HTA). The search was limited to studies of human beings, but no language restrictions were imposed. The search terms included the methodological filters of the EPOC combined with selected MeSH terms (handwashing) and free text terms (hand washing and hand hygiene) as used by Naikoba and Hayward [20]. The search strategies used are outlined in Additional file 1.

### Selection criteria

Studies had to include at least one outcome comparison with a randomised or nonrandomised comparison group, or a comparison with baseline data in the case of a single group before-and-after test design. Other criteria were:

1. Population: HCWs in hospital settings

2. Intervention: strategies aimed at improving HH behaviour

3. Comparison: HH behaviour before the introduction of the programme or strategy, or HH behaviour in a comparison group where another programme or no programme (usual care) was implemented

4. Outcome: all operationalisations of HH behaviour of HCWs.

### Selection of articles

Two reviewers (AH and TvA) independently reviewed the titles and abstracts of citations generated by the search to assess their eligibility for further review based on the selection criteria, and chose relevant articles for possible inclusion. Differences in selection were resolved by consensus or consultation with a third reviewer (MH or LS) in cases of doubt. From potentially eligible studies, the full text papers were subjected to the same evaluation strategy.

### Quality assessment

Rather than exclude studies deemed *a priori* to be of poor quality, we chose to include such studies and empirically rate the level of quality. We used a rating system adapted from Anderson and Sharpe [30], who evaluated the impact of various types of interventions on behaviour change directed either at patients or HCWs. (see Table 2).

**Table 2:** Methodological quality rating


**Design of study or assignment rating**	
Experimental: RCT, random allocation; CCT, quasi-random allocation; three data collection points before and after the intervention	1
Quasi-experimental: CBA, comparable control sites	1
Quasi-experimental: nonequivalent control sites	0
Single group before-after tests with baseline measurement	0
**Content**	
Intervention is clearly described	1
**Sample size**	
Described and justified. An n per group sufficient to detect a significant effect (p < 0.05) with a power of 0.80 or reported calculation of power	1
**Validity and reliability of instruments**	
Unobtrusive observations, rater procedure described and *r* > 0.80	2
Unobtrusive observations, rater procedure not described or *r* < 0.80	1
Obtrusive observations, rater procedure described and *r* > 0.80	1
Obtrusive observations, rater procedure not described or *r* < 0.80	0
Volume of soap or hand alcohol used	0
**Test statistics**	
Test statistics are described	1
**Significance**	
p Value or confidence interval is given	1

CBA = controlled before-and-after study, CCT = controlled clinical trial, ITS = interrupted time series.

The quality rating is a modification of Anderson and Sharpe’s [30] rating.

Two reviewers (AH and TvA) independently determined whether studies met the criteria set for methodological quality, and disagreements were again resolved by discussion. Studies with less than three out of seven points were removed. Studies that rated three points but failed to have a positive score for ‘instruments used’ were removed. Studies that rated three (with a positive score for ‘instruments used’) to five points were graded as moderate quality, and those with six or seven points were graded as high-quality studies.

### Data extraction and synthesis

We used a two-step approach to examine the studies. First, we extracted the study characteristics using the EPOC Data Collection Checklist that includes study objectives, setting, study design, target population, outcome measures, description of the intervention, analysis, and results [31]. Second, to determine which improvement activities could be considered as behavioural change techniques targeting important determinants of adherence behaviours, we used a pre-structured form including the taxonomy of behavioural change techniques of De Bruin *et al.*[26]. Although the taxonomy has been primarily applied in health promotion research, we consider this taxonomy as a valuable tool for in-depth evaluation of HH improvement strategies because these strategies are also aimed at changing behaviour of HCWs. The taxonomy used is an adapted version of the 26-item taxonomy developed by Abraham and Michie [27]. Whereas the original taxonomy already provides a list of well-defined techniques for behaviour change, it was further developed and adapted by De Bruin and colleagues who categorised the behaviour change techniques according to the determinants of behaviour they address. The taxonomy thus provides nine categories to distinguish between techniques addressing knowledge, awareness, social influence, attitude, self-efficacy, intention, action control, maintenance, and facilitation. These determinants are derived from an integration of theoretical constructs from prevailing behaviour (change) theories that have been found predictive of a range of different health behaviours [28]. Together, the nine categories of determinants include a total of 38 behaviour change techniques. See Table 3 for a selection of the most relevant techniques with this overview.

**Table 3:** Selection* of the most relevant techniques and their determinant with this overview

**Determinant**	**Behaviour change technique**	**Description of the activity in studies**
**Knowledge**	Provide general information	Educational sessions or educational materials
	Increase memory or understanding of information	Group discussion, answering questions, clarification
**Awareness**	Risk communication	Information about risks of non adherence or inadequate hand hygiene (infection rates, costs)
	Delayed feedback of behaviour	Overview of recorded hand hygiene behaviour
	Direct feedback of behaviour	Using a system to make professionals aware of their hand hygiene behaviour soon after planned execution
	Feedback of clinical outcomes	Overview of nosocomial infections
**Social influence**	Provide information about peer behaviour	Information about peers’ opinions of correct hand hygiene
	Provide opportunities for social comparison	Group sessions with peers in which discussion and social comparison of hand hygiene practices can occur
	Mobilise social norm:	Exposing the professional to the social norm of important others (not peers) such as opinion leaders
**Attitude**	Persuasive communication	Positive consequences of proper hand hygiene
	Reinforcement of behavioural progress	Praise, encouragement, or material rewards
**Self-efficacy**	Modeling	Use of a role model. Demonstration of proper hand hygiene behaviour in group, class, or team
	Verbal persuasion	Messages designed to strengthen control beliefs about the way of performing correct hand hygiene
	Guided practice	Teaching skills and providing feedback. Specific instruction for correct hand hygiene behaviour
	Plan coping responses	Identification and coping with potential barriers
	Set graded tasks, goal setting:	Desired hand hygiene behaviour is achieved with a stepwise model
**Intention**	General intention information	Explanation of the goals and targets concerning hand hygiene
	Agree to behavioural contract	Contract or commitment with formulated goals of hand hygiene behaviour
**Action control**	Use of cues	Reminders
**Maintenance**	Following behavioural change	Not addressed
**Facilitation of behaviour**	Provide materials to facilitate behaviour	Supportive materials are provided for the healthcare workers
	Continuous professional support	Involves service provided by infection control team or working group, and/or an additional nurse who attends the implementation

* Only terms and definitions for techniques identified in the studies on promoting hand hygiene in healthcare workers are presented.

All reviewers participated in a four-hour training on identifying and coding behavioural techniques in line with the taxonomy. A coding manual (Additional file 2) guided the training. This manual contained comprehensive and detailed criteria for assessing the behaviour change techniques and their related determinants. These criteria and any ambiguities were discussed during the training. Then, we performed a pilot using three excluded studies to validate our scoring results. Finally, two pairs of reviewers (AH and TvA or LS and MH) used the taxonomy to independently code the complete range of improvement activities in the included studies into behaviour change techniques. The techniques identified were grouped under their related determinant. The same procedure was also applied to code ‘usual’ or ‘standard’ care provided to control groups The reviewers who coded the strategy were blinded for the study results, and vice versa. Differences in coding (*i.e.*, <5%) were resolved through discussion. See Additional file 3 for a worked example of data extraction and coding.

### Data analysis

Given the heterogeneity of the studies with regard to target groups, content and delivery of strategies, and opportunities/moments for HH, no formal meta-analysis was done. We describe frequently used strategies at the level of the nine categories of determinants within the classification of the Taxonomy of Behavioural Change Techniques by reporting the frequency with which the determinants were addressed across all studies included in this review.

We analysed the effectiveness at the level of the nine categories of determinants and compared studies addressing one or more determinants. To obtain methodological soundness, we only make inferences about effectiveness using data of the controlled studies (*i.e.*, randomised controlled trials, controlled before-and-after studies, and studies with a cross-over design).

The overall effect size was determined by calculating the relative difference between the intervention and control groups in each controlled study. This relative difference represents the ratio of difference (in percentages) between the interventional and control groups. We obtained the value by dividing the difference between the post-intervention performance scores from the interventional and control groups by the post-performance test scores of the control group, multiplied by 100 (see Additional file 4). To combine findings across studies, we computed the median effect size and the range, representing the results of strategies related to determinants. We decided to report the median because it is less sensitive to extreme scores and provides a better estimate of what the ‘average’ is. Most of the studies included in this review evaluated short-term effects, so we only report results derived from measurements made directly after the interventions were completed.

## Results

**Figure 1:**
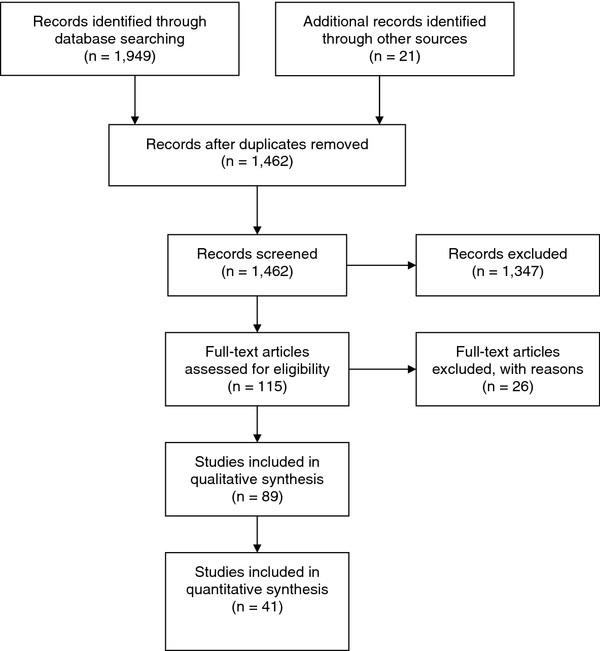
Flow diagram for study selection.

Our search of published works from 2000 through 2009 resulted in 1949 hits for all the databases. A total of 119 studies met the inclusion criteria, including the 21 studies that Naikoba and Hayward reviewed. We assessed the full text of 115 studies (the full text of four studies could not be retrieved). Twenty-six studies were excluded, mostly because of the absence of HH compliance outcomes or studies were non-interventional. In the initial review, 89 studies appeared potentially eligible for review and were read in detail. After quality assessment, 41 studies were included for analysis, and 48 studies were excluded due to major quality limitations, including 10 studies previously reviewed by Naikoba and Hayward (Figure 1). See Additional file 5 for characteristics of excluded studies.

### Study characteristics

Additional file 6 provides an overview of study characteristics in the 41 studies reviewed. Naikoba and Hayward had previously reviewed 11 studies that were published from 1986 through 1999, and the remaining 30 studies were published from 2000 through 2009. Twenty-eight studies had a before-after test design, seven had a controlled before-after design, three were randomised controlled trials, and three had a cross-over design. The study settings were predominantly intensive care units (n = 25), followed by medical or surgical wards (n = 10), emergency wards (n = 4), and 2 studies covered all hospital wards. Multicentre trials were conducted in three studies (two to four hospitals) and the number of participating wards varied from one to three per hospital. In 28 studies, the target population was specified as nurses, physicians, and other HCWs. Six studies targeted only nurses, while seven studies did not specify the type of HCW. The unit of analysis was defined as HH opportunities or moments for HH (n = 33), participants (n = 5), patients (n = 1) and number of dispenser activations (n = 2). Most studies (n = 39) reported HH compliance rates as a primary outcome measure. These data were collected by means of unobtrusive observations (n = 30) or by obtrusive observations (n = 9) in HCWs. One study measured HH performance by volume of soap and hand alcohol used, and one study identified HH episodes by using an electronic counting device. Six studies based their strategy on barriers identified by practice research such as skin irritation, workload, staff personal habits, and priorities. Eleven studies mentioned barriers derived from the literature. The rating of study quality resulted in six high-quality studies. Each of these studies scored six points on our rating scale. Two of the moderate-quality studies scored three points, 28 studies scored four points, and five studies scored five points. Identified quality limitations were: uncontrolled study design (n = 28), absence of sample size justification (n = 33), observations without a description of inter-rater reliability agreement (n = 31), and no description of test statistics (n = 3).

### Determinants addressed (n = 41)

**Figure 2:**
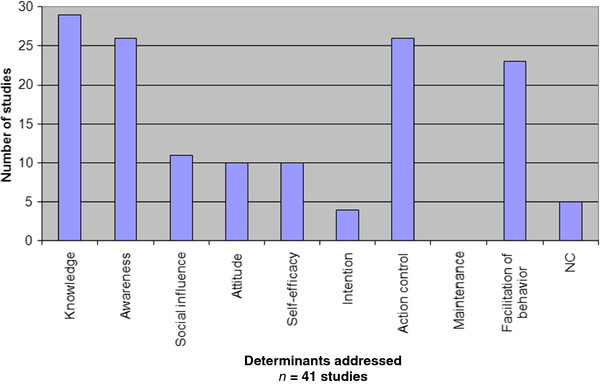
**Numbers of studies addressing specific determinants of behaviour change.** Knowledge (29), Awareness (26), Social influence (11), Attitude (10), Self-efficacy (10), Intention (4), Action Control (26), Maintenance (0), Facilitation of behaviour (23), NC = no coding possible (5). Total = 144 in 41 studies.

We evaluated the HH improvement strategies across the controlled and uncontrolled studies Figure 2 shows the number of studies addressing specific determinants. The most frequently addressed determinants were knowledge (n = 29), awareness (n = 26), action control (n = 26), and facilitation of behaviour (n = 23). Fewer studies addressed social influence (n = 11), attitude (n = 10), self-efficacy (n = 10), and intention (n = 4). One determinant directed at behavioural maintenance following behaviour change was not addressed at all. Five studies used techniques focused mainly on gaining senior management support and commitment, and institutional priority for HH [32-36]. These activities could not be coded because they were primarily directed at gaining support for program implementation rather than serving as a technique to change HH behaviour directly.

The 14 studies that addressed one or two determinants mainly consisted of combinations of knowledge, awareness, action control, and facilitation of behaviour (Table 4). Only one study in this group added social influence to its strategy [37]. Moongtui combined social influence with awareness. Colleagues evaluated each other’s performance on appropriate hand washing and glove wearing. The investigators also provided feedback at group level by posting compliance scores anonymously on a bulletin board every three days.

**Table 4:** Content of strategies related to determinants of behaviour change

**Studies n = 41**	**Determinants of behaviour change [studies]**
**9**	**Studies addressing one determinant** (3 controlled and 6 uncontrolled studies)
2	Action control [38]*; [39]
2	Awareness [40]*; [41]
5	Facilities [42]; [43]; [44]; [45]*; [46]
**5**	**Studies addressing two determinants** (1 controlled and 4 uncontrolled studies)
2	Knowledge, Action control [47]; [48]
1	Knowledge, Facilities [49]
1	Awareness, Action control [50]
1	Awareness, Social influence [37]*
**8**	**Studies addressing three determinants** (3 controlled and 5 uncontrolled studies)
2	Knowledge, Awareness, Action control [51]; [52]
1	Knowledge, Awareness, Facilities [53]*
1	Knowledge, Awareness, Attitude [54]
1	Knowledge, Awareness, Self-efficacy [55]*
2	Knowledge, Action control, Facilities [56]*; [57]
1	Knowledge, Action control, Intention [58]
**6**	**Studies addressing four determinants** (2 controlled and 4 uncontrolled studies)
2	Knowledge, Awareness, Facilities, Action control [59]; [60]
1	Knowledge, Awareness, Facilities, Social influence [35]*
1	Knowledge, Self-efficacy, Action control, Awareness [61]
1	Knowledge, Self-efficacy, Action control, Facilities [62]
1	Self-efficacy, Intention, Awareness, Social influence [63]*
**9**	**Studies addressing five determinants** (3 controlled and 6 uncontrolled studies)
2	Knowledge, Awareness, Action control, Social influence, Attitude [64]; [65]
2	Knowledge, Awareness, Action control, Social influence, Facilities [1]*; [66]
2	Knowledge, Awareness, Action control, Facilities, Attitude [67]*; [68]
1	Knowledge, Awareness, Facilities, Attitude, Self-efficacy [69]*
1	Knowledge, Awareness, Facilities, Self-efficacy, Action control [32]*
1	Knowledge, Facilities, Self-efficacy, Action control, Attitude [34]
**1**	**Studies addressing six determinants** (1 uncontrolled study)
1	Knowledge, Awareness, Social influence, Attitude, Action control, Facilities [33]
**3**	**Studies addressing seven determinants** (1 controlled and 2 uncontrolled studies)
1	Knowledge, Awareness, Social influence, Self-efficacy, Intention, Action control, Attitude [70]*
1	Knowledge, Awareness, Social influence, Self-efficacy, Intention, Action control, Facilities [71]
1	Knowledge, Awareness, Social influence, Self-efficacy, Action control, Attitude, Facilities [36]

* Controlled study.

Fourteen studies addressed three or four determinants and used combinations as described above, but seven studies also addressed determinants as social influence, attitude, self-efficacy, or intention. For example, Huang focussed on increasing knowledge (educational training programme and written information) and awareness (clarifying risks for blood pathogen exposure), but also enhanced the self-efficacy of nurses with one hour of practical demonstration of hand washing and using gloves [55]. In Marra’s study, activities were also aimed at increasing awareness by providing feedback on infection rates. The nurse manager also provided opportunities for social comparison by showing each HCW the total number of times the dispensers were used in each patient room in which the HCW worked compared to the number of times that other HCWs used dispensers. In addition, the nurse manager explained the goals and targets of the HH improvement strategy twice a week, thus strengthening intention and self-efficacy [63].

All 13 studies addressing five or more determinants consisted of activities addressing multiple different determinants. For example, Trick *et al.* addressed determinants such as knowledge (educational sessions and distribution of educational materials to professionals), awareness (displaying HH adherence), action control (hospital-wide poster campaign), facilities (alcohol-based hand rub), and attitude (pointing out the benefits of using alcohol-based hand rubs) [67].

We found no differences in the extent to which determinants were targeted between the controlled studies and uncontrolled studies (Table 4).

See Additional file 7 for details of improvement activities and results in the 41 studies reviewed.

### Effectiveness

Table 5 presents the effectiveness of the controlled studies related to their determinants of behaviour change. Controlled studies addressing one determinant focussed on action control (n = 1), awareness (n = 1) or facilitation of behaviour (n = 1). The median effect for these strategies was a relative difference (improvement) of 17.6 in performance. The effect size from one controlled study addressing two determinants was 25.7. The relative difference increased from 42.3 in the three studies addressing three determinants to 43.9 for the two studies addressing four determinants. The relative difference was 49.5 for the three studies that addressed five determinants.

**Table 5:** Effectiveness of controlled studies related to determinants of behaviour change

**Determinants of behaviour change [studies]**	**Effect size**
**All studies (n = 13)**	**R = relative difference between intervention and control**^**$**^
	**M = median [range]**
	**25.7 [-8.8 to 429]**
**Studies addressing one determinant**	n = 3
	M: 17.6 [-8.8 to 61]
Action control [38]	n = 1
	R: -8.8
Awareness [40]	n = 1
	R: 17.6
Facilities [45]	n = 1
	R: 61.0
**Studies addressing two determinants**	n = 1
	M: 25.7 [25.7*]
Awareness, Social influence [37]	n = 1
	R: 25.7
**Studies addressing three determinants**	n = 3
	M: 42.3 [19.5 to 82.7]
Knowledge, Awareness, Facilities [53]	n = 1
	R: 19.5
Knowledge, Awareness, Self-efficacy [55]	n = 1
	R : 42.3
Knowledge, Action control, Facilities [56]	n = 1
	R: 82.7
**Studies addressing four determinants**	n = 2
	M: 43.9 [14.8 to 73*]
Knowledge, Awareness, Facilities, Social influence [35]	n = 1
	R: 73
Self-efficacy, Intention, Awareness, Social influence [63]	n = 1
	R: 14.8
**Studies addressing five determinants**	n = 3
	M: 49.5 [-8.6 to 429]
Knowledge, Awareness, Action control, Facilities, Attitude [67]	n = 1
	R: 49.5
Knowledge, Awareness, Facilities, Attitude, Self-efficacy [69]	n = 1
	R: -8.6
Knowledge, Awareness, Facilities, Self-efficacy, Action control [32]	n = 1
	R: 429
**Studies addressing seven determinants**	n = 1
	M: 9.7 [9.7 *]
Knowledge, Awareness, Social influence, Self-efficacy, Intention, Action control, Attitude [70]	n = 1
	R: 9.7

*Median and range calculated over fewer than three studies.

^$^Relative difference calculated as (the results from the intervention group after the intervention minus the results from the control group after the intervention) divided by the results from the control group after the intervention.

No controlled study addressed six determinants. The only controlled study addressing seven determinants showed less impact on short-term effectiveness (relative difference 9.7). However, baseline HH rates in this study were higher in the intervention group than in the control group, probably because administrators were already planning and discussing the strategy during the baseline phase [70].

**Figure 3:**
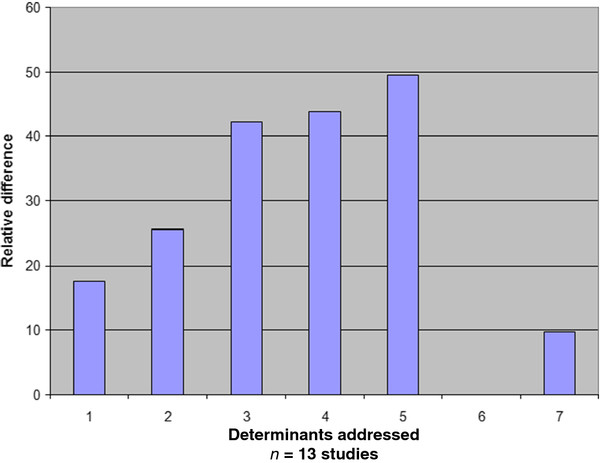
**Correlation effectiveness and determinants addressed.** Pearson correlation coefficient r = 0.961; p = 0.00.

The increase in effectiveness correlated closely with the number of determinants (one to five) addressed (Pearson’s correlation coefficient = 0.961, p = 0.009) See Figure 3.

## Discussion

Improved HH behaviours among hospital personnel could have a considerable impact on HAIs, healthcare costs, and patients’ health and quality of life. Yet, reviews with detailed examination of the active content of strategies to promote HH are missing. In the present study, the content and effectiveness of a range of strategies to improve the HH adherence of HCWs were studied. By using a detailed coding taxonomy of behaviour change techniques targeting major behavioural determinants, we were able to obtain a detailed insight into frequently used HH improvement strategies and how they work. Analysing the content of the strategies at the level of determinants that prompt HH behaviour, it was found that those studies focusing on combinations of different determinants gave better results, which indicates that we should be more creative in the application of alternative improvement activities aimed at altering specific behavioural determinants change, such as social influence, attitude, self-efficacy, and intention.

Although the content of the strategies and related determinants varied greatly, most of the studies addressed more than one determinant (mainly knowledge, awareness, action control, and facilitation of behaviour). This is consistent with Naikoba and Hayward’s findings and previous systematic reviews of changing professional behaviour in which education (addressing ‘knowledge’), feedback (addressing ‘awareness’), reminders (addressing ‘action control’), and facilities (addressing‘facilitation of behaviour’) were the most frequently used improvement activities [12,20,21]. Twenty strategies addressed additional determinants that prompt HH behaviour such as social influence, attitude, self-efficacy, or intention. These specific determinants were especially targeted in comprehensive strategies that addressed at least four determinants. This provides new insight into the content of HH improvement strategies: half of the studies used a strategy targeting determinants not mentioned in previous reviews of HH adherence.

Most studies addressed determinants at the individual and institutional levels; specific team-oriented activities were hardly identified. Strategies including team-directed activities could, however, be valuable because HCWs (especially nurses) usually work in teams. Evidence for the effectiveness of team-directed strategies in other settings exists, but these strategies are rarely applied in studies of HH improvement [72,73]. Surprisingly, activities directed at behavioural maintenance following behaviour change were not identified in the studies. Nonetheless, activities aimed at persistence should be part of the strategy for achieving sustainability of improved HH behaviour.

The effectiveness of the strategies varied substantially, but most controlled studies showed positive results. This is in line with previous review findings [74,75]. If determinants such as social influence, attitude, self-efficacy, and intention are targeted within a strategy, the effect is larger than that of strategies consisting solely of a combination of determinants, such as knowledge, awareness, action control, and facilities. Apparently, these specific determinants provide an additional contribution to effectiveness. This finding is confirmed by results of previous studies where social influence, attitude, self-efficacy, and intention are considered relevant to successfully changing behaviour [26-29].

The median effect size increased when more determinants were addressed. In other words, there seems to be a dose response effect. This result deviates from Grimshaw *et al.*’s finding that there was no dose response relation between the number of improvement activities and the effects of multifaceted strategies [75]. The lack of a rationale in the composition of a multifaceted strategy, such as mentioned by Grimshaw, may be a good explanation for the lack of a relationship between the number of improvement activities and the effect. An additional explanation for this discrepancy can be found in the framework chosen to classify the strategies for change. Grimshaw used the EPOC classification of strategies that is based on the form of performed improvement activities. We used an alternative approach that classed improvement activities on the basis of their determinants of behaviour change. By using the Taxonomy of Behavioural Change Techniques we collected information about triggers that encourage behaviour change rather than describing separate improvement activities. Thus, using multiple activities is not necessarily the same as addressing multiple determinants or vice versa. For example, the combined distribution of educational materials and provision of educational sessions constitute two different improvement activities. We would not label this strategy as multifaceted because both activities apply the same determinant (‘knowledge’).

Although we found a maximum effect in addressing five determinants, we cannot provide a ‘one-size-fits-all’ recipe for building a successful strategy. Previous recommendations from the literature have pointed out that an improvement strategy for HH behaviour should address existing problems and barriers [12,73,75]. Analyses of barriers and facilitators and linking improvement activities to these influencing factors are important steps in the design of a strategy and may be crucial to success. A multifaceted strategy with many improvement activities that are not precisely tuned to the existing barriers apparently misses the target; part of the components may be redundant or ineffective. For example, if there is no knowledge shortage, educational strategy components probably will not contribute to the effectiveness of the multifaceted strategy. Barriers also exits at other levels than the individual HCW. Barriers like negative role models, a poor social culture, and disinterested management can hamper good HH. Overcoming these barriers requires the use of alternative activities such as social influence, attitude, self-efficacy, or intention. Of particular interest is the HELPING HANDS study, currently performed in the Netherlands [76]. In this study, improvement activities are directed at gaining active commitment and initiative of ward management; modelling by informal leaders at the ward; and setting norms and targets within the team. This team-directed strategy goes beyond individual and institutional only approaches, but rather addresses determinants at team level by focussing on social influence in groups and strengthening leadership.

In this review, it was not possible to check for this ‘appropriateness’ of determinants addressed within the studies because context and barrier analysis and the rationale regarding strategy selection were hardly reported. Therefore, for most of the studies, it was unclear how well the strategy fitted the context. In view of the effectiveness, but also feasibility and costs, we propose selecting appropriate determinants rather than addressing all determinants.

**Figure 4:**
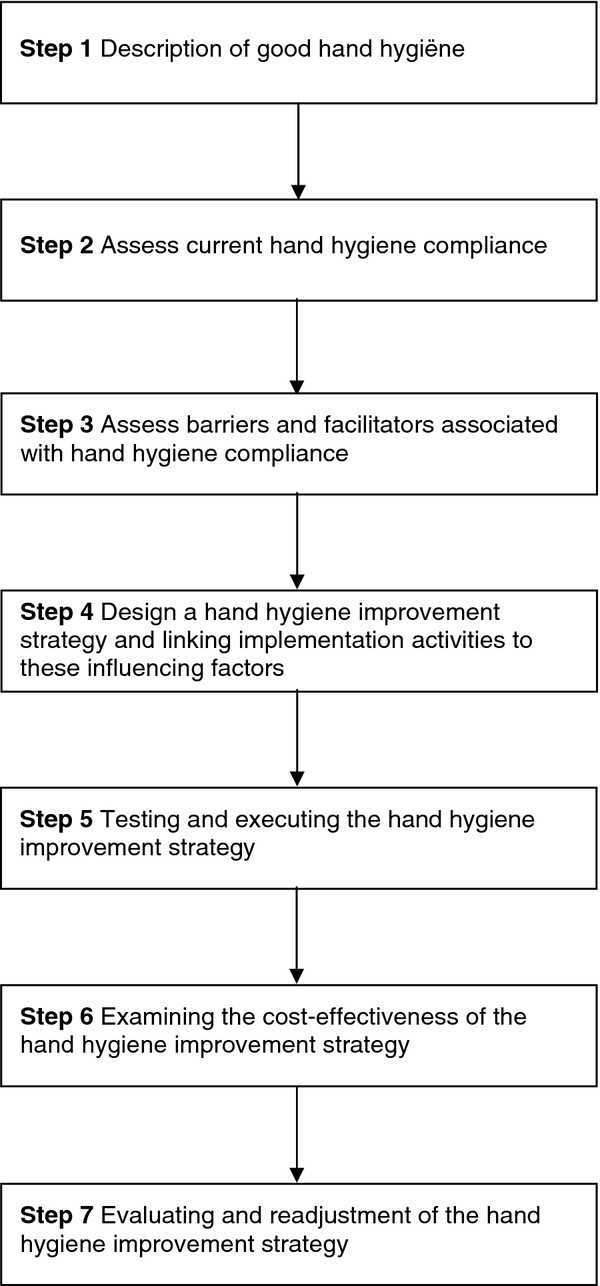
Building a successful hand hygiene improvement strategy.

We concentrated on determinants within strategies—an alternate view, yet crucial to understanding the working mechanism of strategies to improve HH adherence. We were able to identify less commonly addressed determinants, such as social influence, attitude, self-efficacy, and intention, that considerably contribute to the effectiveness of strategies. Our study findings fit well within the implementation model of Grol and Wensing [73] for building a successful HH improvement strategy (see Figure 4).

The Taxonomy of Behavioural Change Techniques was a valuable tool that led us to convert descriptions of improvement activities into well-defined determinants. We obtained a clear focus on theory-based determinants of behaviour change that were hidden in the improvement strategies. We consider this a crucial step in developing a theoretical understanding of the effectiveness of improvement strategies.

## Methodological discussion

Although we succeeded in achieving substantial insight into the content and effectiveness of HH improvement strategies, some aspects should be considered further. First, the methodological weakness of the studies is still a major concern. Most of the studies were small scale; they lacked a control group comparable to the test group, and made no formal attempt to minimise bias. There is a risk that a positive relationship between the number of determinants targeted and the effect on HH compliance might be partly explained by an unknown confounder. This holds particularly true for the observational studies where wards were selected to receive an improvement strategy. In our review, we included studies that clearly described the content of the strategy and were at least of moderate quality. With methodological soundness in mind, we only used results from controlled studies when we reported effectiveness. However, the risk of confounding should be taken into account when interpreting our results. Methodologically robust research is still required to evaluate the effectiveness of interventions intended to improve HH compliance. Adequately powered cluster randomised trials or well-designed ITS studies would provide the optimal study design.

Second, our search included literature up to November 2009. Therefore, we cannot provide information on recently performed HH improvement studies The screening and analysis of the search results as reported in this review served as a starting point for the development of two HH improvement strategies, which were subsequently tested in a randomised controlled trial. The design of this study was published in 2011 [76].

Third, as in any systematic review of the literature, there may be publication bias. Most studies showed positive results; it is possible that studies with negative results have not been published. In our review we were unable to retrieve four articles; it is possible that they contained relevant data.

Fourth, the criteria used to determine when HH should be performed varied over the studies and were not always explicitly stated. This may have implications for the generalisability of the results of the studies.

Fifth, good reliability in coding the improvement activities was observed (>95%), suggesting that our instructions and definitions can be applied reliably after only brief training. Within all steps of the review process, validity was increased by using standardised methods and forms as well as multiple raters. However, once techniques and targeted determinants are well chosen, examining the actual exposure to the improvement activities was problematic. Studies did not or marginally report on how well the improvement strategy was implemented. Designating HH as hospital goal, for example, requires setting specific, realistic, and measurable targets [77]. However, descriptions of the improvement activities in the studies provide insufficient detail to check for appropriate delivery as well as the actual exposure of the HCWs to this activity. Without sufficient information about implementation fidelity, it is hard to determine whether the impact of the HH improvement strategy is due to the implementation process or to the composition of the strategy itself, a so-called Type III error [78].

Finally, most studies did not describe, or only marginally described, the activities of the ‘usual’ or ‘standard’ care provided to control groups. Standard care practices may vary from site to site. Therefore, describing standard care is important for the interpretation and comparison of intervention effects. Given the combination of strengths and considerations, this review provides an original and valuable overview of various strategies for improving the HH adherence of HCWs.

## Conclusions and future directions

By focussing on determinants of behaviour change, we found hidden and valuable components in HH improvement strategies. Addressing only determinants such as knowledge, awareness, action control, and facilitation is not enough to change HH behaviour. Addressing combinations of different determinants provided better results. This indicates that we should be more creative in the application of alternative activities addressing determinants such as social influence, attitude, self-efficacy, or intention.

A systematically designed strategy that targets various problems and barriers to change, with activities at different levels (professional, team, and organisation), is needed to achieve changes in HH behaviour. Currently, most strategies focus on the individual and the organisation, while group- or team-directed strategies are rarely used. Including team-directed techniques in a strategy is a promising development.

## Competing interests

The authors declare that they have no competing interests.

## Authors’ contribution

All authors conceived and designed the study, drafted and revised the manuscript, and approved the final version. AH performed the literature search. MdB, TvA, and AH discussed and assessed the application of the taxonomy of behaviour change techniques. AH TvA, MH, and LS selected articles for inclusion and also analysed and interpreted the data. AH wrote the first draft of the manuscript. TvA, MH, LS, MdB and RG critically reviewed and edited revised the manuscript.

## Source of funding

This study is funded by a research grant from ZonMw, dossier number: 94517101.

## Supplementary Material

Additional file 1 Search strategy by database. Click here for file

Additional file 2 Coding manual. Click here for file

Additional file 3 Worked example data extraction. Click here for file

Additional file 4 Calculation of relative difference. Click here for file

Additional file 5 Characteristics of excluded studies. Click here for file

Additional file 6 Overview of strategies and methods in the 41 studies reviewed. Click here for file

Additional file 7 Details of study characteristics, improvement activities and results in the 41 studies reviewed. Click here for file
