# Supplementary material for: A systematic review of hand hygiene improvement strategies: a behavioural approach
Source: Implement Sci. 2012 Sep 14;7:92. doi: 10.1186/1748-5908-7-92 (PMC3517511; doi:10.1186/1748-5908-7-92)
Supplement: Additional file 1 — Search strategy by database. [file 1748-5908-7-92-S1.pdf]

Additional file 1: search strategy by database

Database: Ovid MEDLINE(R) <1950 to November Week 3 2009>
Search Strategy:
----

1 Randomized controlled trial/
2 random$.tw.
3 experiment$.tw.
4 (time adj series).tw.
5 (pre test or pretest or post test or posttest).tw.
6 impact.tw.
7 intervention$.tw.
8 chang$.tw.
9 evaluat$.tw.
10 effect?.tw.
11 compar$.tw.
12 control$.tw.
13 or/1-12
14 Nonhuman
15 13 not 14
16 (hand washing or handwashing or hand hygiene).
17 16 and 15
18 limit 17 to yr="2000 - 2009"

Database: EMBASE <1980 to 2009 Week 48>
Search Strategy:
----

1 Randomized controlled trial/
2 random$.tw.
3 experiment$.tw.
4 (time adj series).tw.
5 (pre test or pretest or post test or posttest).tw.
6 impact.tw.)
7 intervention$.tw.
8 chang$.tw.
9 evaluat$.tw.
10 effect?.tw.
11 compar$.tw.
12 control$.tw.
13 or/1-12
14 Nonhuman/
15 13 not 14
16 (hand washing or handwashing or hand hygiene).
17 16 and 15
18 limit 17 to yr="2000 - 2009"

Database: CINAHL <1980 to November Week 4>
Search Strategy:
----
1 clinical trials/
2 control$.tw.
3 random$.tw.
4 comparative studies/
5 experiment$.tw.
6 (time adj series).tw
7 impact.tw.)
8 intervention$.tw.
9 evaluat$.tw.
10 effect?.tw.
11 exp pretest-posttest design/
12 exp quasi-experimental studies/
13 or/1-12
14 (hand washing or handwashing or hand hygiene).
15 13 and 14
16 limit 15 to yr="2000 - 2009"
