# Supplementary material for: A systematic review of hand hygiene improvement strategies: a behavioural approach
Source: Implement Sci. 2012 Sep 14;7:92. doi: 10.1186/1748-5908-7-92 (PMC3517511; doi:10.1186/1748-5908-7-92)
Supplement: Additional file 2 — Coding manual. [file 1748-5908-7-92-S2.pdf]

Coding manual for behavioral change techniques

An adapted version of the coding manual from Abraham C & Michie S. A taxonomy of behavior change techniques used in interventions: The Coding Manual. (2007). Available from the authors: s.c.s.abraham@sussex.ac.uk

Important additional source was: Bartholomew LK, Parcel GS, Kok G, Gotlieb NH. *Planning health promotion programs*. San Francisco, CA; Jossey-Bass:2006. (Mainly chapters 3, 4, and 7).

M. de Bruin, G. Kok, H. Schaalma & H. Hospers (2007).
Maastricht University, Faculty of Psychology
Department of Work & Social Psychology

Corresponding author: Mar.debruin@psychology.unimaas.nl

General instructions

Please carefully read the taxonomy before coding materials for behavioral change techniques. Discuss the techniques with co-coders to make sure that these are interpreted similarly by all coders. Always practice coding on practice materials comparable to your final materials and discuss these coding results before starting actual coding.

Suggestions for optimal coding (for coders individually):

- Read the [coding material] once before actual coding. Highlight relevant sections.
- Scan the different techniques presented in the coding table (last page).
- Start coding the relevant sections using the scoring table. In case of any doubt between techniques, always turn to the description of the techniques presented in this document.
- Provide each coded activity with a number in the original coded material (e.g. #4) that you also write behind the relevant technique in the coding table (e.g. Persuasive Communication: #4).
- There are 4 coding columns in the coding table. Two for the first intervention contact(s), two for follow-up contacts. The white column is to code techniques about which the coder is (relatively) sure, the grey section to note techniques about which the coder is unsure and may want to get back to later. Decide before comparing inter-coder reliability scores what to do with these different categories of (un)certainty: Ignore or include the grey section.
- Two techniques, i.e. Self-report of behavior and Electronic monitoring of behavior, are no actual behavioral change techniques. Coding of these techniques can be informative but should not add to a score for intervention quality. Since these techniques are linked with other techniques, they are included in this taxonomy list.

After you have finished coding the materials, please review the completed coding table to make sure that

- You have scored the correct techniques
- The techniques you have **not** scored are definitively not used in the intervention (e.g., you can cross out techniques that you think have not been scored).

Tips:

- Print this document with 2 pages per sheet, 1-sided, so that you only have 3 pages with detailed techniques on your desk.
- The coding table is on A3 but is readable on A4 (print à Scale to paper Size à A4).

Request: If you have suggestions for improvements or extensions of this coding manual, please contact the corresponding author. This manual is a work in progress and will be adapted over time.

NB. The explanations of techniques in this taxonomy are applied to adherence to HIV-medication. These illustrations can easily be adapted to fit other treatments and topics: The behavioral change techniques remain the same.

NB. Coding intervention materials provides information about what techniques were *intended* to be delivered (intended intervention quality). This need not correspond with what techniques were actually delivered to patients. Coding actual intervention sessions or descriptions of activities that were actually executed would lead to more accurate coding of intervention quality. What may help are implementation evaluations, evaluations of what parts of the intended intervention was adequately delivered.

General techniques

General techniques are techniques that can be an addition to the Specific techniques (described on the following pages). In case a general technique is applied to a specific technique, it should be scored in addition to the specific technique. In case a general technique is an addition to a specific technique, this is mentioned in the definition of the specific technique and in the coding table.
There are two exceptions, namely 2a: Tailoring of the whole intervention protocol based on participant characteristics measured beforehand, and 2b: Tailoring of the number of visits to the need of the participant. These are “macro/meso”-tailoring techniques, not containing or applied to a specific technique, and are therefore considered a separate category. **On the bottom of the scoring table (final page these techniques can be coded and described separately.**

The general techniques are:

1. Individualization
The provision of opportunities for learners to have personal questions answered or instructions paced according to their individual progress. Merely the opportunity to ask questions because there is a 1-on-1 contact is not sufficient to score this technique: the person must be either prompted to ask questions and/or it must be described that, and how, instructions are paced according to individual progress.

2. Tailoring
Adapt the complete intervention strategy **or** specific intervention components to previously measured characteristics of the person. Score tailoring at the following different levels (see scoring-table):

a) Macro-tailoring (group level): The intervention that the person receives depends on certain pre-tested characteristics. E.g. a different intervention is applied to persons in different motivational stages, or the type of intervention depends on the level of adherence.
b) Meso-tailoring (individual level): The amount of intervention contacts depends on the needs of the patient, e.g. someone with complex adherence problems would return after 4 weeks instead of after 12 weeks.
c) Micro-tailoring (individual level): Specific techniques are tailored to the patient: e.g. action plan tailored to individual’s lifestyle; risk information tailored to person’s risk status.

NB These techniques can all be used in one intervention: it is possible to tailor the intervention group characteristics (motivational stage), tailor the number of intervention contacts to individuals’ needs and problems, and tailor specific techniques.

3. Participation
The basic approach of an intervention can be to have the participant actively involved in various stages of the intervention, i.e. determine causes of non-adherence, determine how to change behavior, decide on behavioral targets, evaluate outcomes, etc. In case the intervention description suggests that a dialogue should take occur, is would be an instance of this technique. Participation logically leads to micro-tailoring of techniques, so participation and micro-tailoring may often look similar and can then be scored as any of the two (depends on what category is shown in the coding table under the relevant technique).
NB To score this technique the person must be prompted to provide input or make decisions in relation to relevant techniques.

Specific techniques (short descriptions)

Knowledge

1. Provide General Information.
Basic information about HIV, the medicines, the role of adherence, and how much adherence is enough.
Tailored: Information can be tailored to the current level of knowledge of the person
Individualization: The person is prompted to have personal questions answered.
NB
Information about **Negative consequences** of target behaviors: technique #3 “Risk communication”.
Information about **Positive consequences** of target behaviors: technique #15 “Persuasive communication”
All three techniques can be used concurrently.

2. Increase memory and/or understanding of transferred information.
Use of **images** or **metaphors**, or prompts for **rehearsing** or **repeating** information in own words, **and similar strategies**.
Also **Group discussion:** Prompt participants to ask questions, clarification, and elaboration. Participants must be prompted to do so, merely a group setting is not sufficient. Expert presence is required.
NB
1-on-1 communication (person and professional): prompts for questions, clarification, and elaboration would be an instance of individualization under technique #1 “Provide general information”.

Awareness

3. Risk communication.
Information about costs/risks of action or inaction with respect to target behaviors. Also entails risk-communication strategies such as scenario-based risk information and fear appeals.
Tailored: to the person’s risk status (e.g. current behavior, clinical profile).
NB
Information about **Positive consequences** of target behaviors: technique #15 “Persuasive communication”
Messages **not** including information on + or - outcomes: Technique #1 “General information”.

4. Self-monitoring of behavior
Person keeps a record of specified behaviors. E.g. a diary or a questionnaire of behavior over multiple time points between two intervention contacts (minimum duration e.g. 1 week), or patients makes notes of when and in what situation (s)he experienced problems correctly executing the target behavior.
NB
Different from **Self-report** (#5): assessment of behavior through self-report does not require previous self-monitoring. Always **choose either technique #4 or #5**, not both.

5. Self-report of behavior (!Not an actual change technique, but coding can be informative!)
Without prior instances of self-monitoring (technique #5), the person is asked to self-**report** behavior**.** The self-report should at least concern the last 3 days. An electronic monitoring device that requires pressing a button at every intake, would also count as an instance of self-report.
NB
Different from technique #4 in that self-reports do not involve reporting self-monitored data.

6. Electronic monitoring of behavior (!Not an actual change technique, but coding can be informative!)
Person is asked to monitor medication intake using an electronic monitoring device (**MEMS-cap, SMART-cap**) that automatically records “medication intake”.
NB
If the electronic device requires the person to **press a button to register medication intake**, it would be an instance of technique #5 “Self-report of behavior”.
In case the person uses a **SMART-cap** (or, **MEMS-view cap**) to monitor behavior, this would automatically **also** imply use of technique #9 “Direct feedback on behavior”.

7. Reflective listening: direct feedback of cognitions and emotions
Feedback of cognitions and emotions through reflective statements during **1-on-1** communication with the professional intended to increase awareness of ideas, reasoning and emotions. Scoring requires explicit mention of this technique.
NB
Reflective statements can include reflecting **ambivalence** between important goals and values in life and current behavior. Reflecting ambivalence would be an instance of technique #14 “Reevaluation, self-evaluation”.
Different from techniques #8 and #9 as these concern **feedback of behavior.**

8. Feedback: Delayed feedback of behavior
Includes providing an **overview of recorded behavior.** For that, behavior has to be recorded daily using either technique #4, technique #5-**eletronically** (so not just a 3-day self-report), or technique #6. Code whether behavior was recorded **subjectively** (technique #4 & #5-electronically) or **objectively** (#6).
NB
Difference between #8 (a) & (b) is that the latter only concerns an indication of behavior over the last 3-7 days. Thus these data do not give an overview of behavior.
When feedback is linked to **previously formulated goals** instead of objective reference (#8b), it is an instance of technique #30 and not this technique. But both techniques can be used in one intervention.

9. Feedback: Direct feedback of behavior
Involves **a system** designed to make people aware of their (lack of) behavior (forgetting dose) **soon after** (<24 hours) **planned execution:** e.g. a dosette box with medication organized for every day of the week, or a so-called SMART-cap with a display showing medication container openings per day.
NB
Different from technique #8 that involves a person providing feedback with an overview of recorded data or by providing objective reference of self-reported behavior.
If this technique #9 is used with the purpose to facilitate technique #4, also score that technique.

10. Feedback of clinical outcomes
Concerns feedback provided to the person about clinical outcomes, i.e. CD4 and viral load.
NB
When therapeutic drug levels are fed back, it would be an instance of technique 8(b) and not this technique.

Social influence

11. Provide information about peer behavior (“Peer passive”)
Information about what peers do and think in relation to the target behavior or preparatory behaviors. This can be provided verbally or by using detailed case studies in text or in video.
NB
Different from technique #12 that involves a **group setting**.
Different from technique #13 that involves a social norm of **Important others** instead of a norm from peers.
Not with the purpose of technique #18 “Modeling i.e. to increase self-efficacy. Focus is on providing social reference, not on how to perform a behavior.

12. Provide opportunities for social comparison (“Peer active”)
Group sessions with peers in which discussion and social comparison can occur. Not with the purpose of modeling or transmission of information: focus is on providing social reference for the behavior. Only score this technique in case discussion of adherence and/or preparatory behaviors is prompted, or experiences with these behaviors are shared (personal stories).

13. Mobilize social norm (“Important others”)
Involves exposing the person to the **social norm of important others** in relation to the target or preparatory behaviors. Important others may be family members, partners, friends, but also healthcare professionals (on the condition that they are important, i.e. a valued and trusted expert).
NB
Different from technique #11 and #12 that explicitly focuses on peers, not on important others.

Attitude

14. Reevaluation of outcomes, self-evaluation.
Prompts to go through a process of (re)evaluation of outcomes of current behavior and alternative behaviors, and how these behaviors and outcomes relate to self-identity and/or important goals and values in life. Includes comparison of **desired behavior** with **actual behavior** (self-regulation theory), and **reflections of ambivalence** between current behavior and important goals/values in life (often used in Motivational Interviewing).
Also includes **Environmental reevaluation:** come to realize the impact of ones' behavior on their environment.
NB
In case **actual behavior** (following monitoring or self-report) is compared to **previously formulated behavioral goals**, it would be an instance of technique #27 and not of this technique.

15. Persuasive communication, belief selection
Messages designed to strengthen positive beliefs about the outcome of the target behavior / behavior change, and/or weaken negative beliefs about behavioral change. New beliefs may be induced and/or new information may be offered with the purpose to create new beliefs.
Tailored: beliefs about the target behavior are explored after which the information is tailored to current belief structure.
NB
Different from technique #19 which is aimed at increasing beliefs about self-efficacy.

16. Reinforcement on behavioral progress, provide contingent rewards,
Includes praise and encouragement as well as material rewards, but the reward/incentive must be explicitly linked to the achievement of specified goals. Also includes use of self-reward strategies.
NB
Different from technique #17 in the sense that this technique reinforces **behavioral progress** while technique #17 concerns reinforcements of **motivational progress**.

17. Reinforcement on motivational progress, provide contingent rewards, affirmation,
Includes praise and affirming remarks as well as material rewards following statements of the person indicating movement towards increased motivation or confidence to change. Also includes reinforcement of efforts to actively participate in the intervention program. Affirmation is a core-component of Motivational Interviewing, but scoring requires explicit mention of this technique.
NB
Different from technique #16 in the sense that technique #16 reinforces **behavioral progress** while this technique #17 concerns reinforcement of **motivational progress**.

Self-efficacy

18. Modeling
Involves *showing* the person how to correctly perform a behavior, e.g. by face-to-face demonstration of the behavior by a professional, in a group class, or by showing a role model in a video.
NB
Different from techniques #11 and #12 that focus on providing social reference while this technique aims to show how to correctly perform a behavior.

19. Verbal persuasion
Messages designed to strengthen efficacy/control beliefs related to execution of the target or preparatory behaviors (e.g. information about often-used successful strategies and general tips). New beliefs may be induced and/or new information may be offered to create new control beliefs.
Tailoring: control beliefs about target behavior are explored after which the information delivered is tailored to the person's current belief structure and/or personal situation and/or capabilities.
NB
Different from technique #15 that focuses on persuasive arguments about the outcomes of the behavior to alter attitudinal beliefs.
When tips are used, these must be relevant for the behavior of the individual at that moment. Therefore, presenting a list of tips that may at some time become useful, would not be an instance of this technique but of technique #1 "Provide General Information".
Different from technique #21 that focuses on first analyzing anticipated problems for successful behavioral execution followed by formulation of strategies specifically designed to overcome these barriers.

20. Practice, guided practice
Prompt the person to rehearse the behavior or preparatory behavior various times; OR have the person practice the behavior after which the exercise is discussed and the professional provides feedback.

21. Plan coping responses
Determine potential barriers and ways to overcome these. Barriers may include competing goals in specified situations, i.e. **prioritizing between goals** in favor of the target behavior. May be described as "**problem solving**" and if this is in relation to performance of behavior, then it is an instance of this technique. Prompts to perform **self-regulatory behaviors** can be considered as an instance of planning coping responses but should at least exist of self-monitoring of adherence followed by having the person develop and implement solutions for problems.
__Participation:__ prompt the person to both determine barriers ***and*** ways to overcome these. No participation would look like instructions.
__NB__
Closely related to technique #26 and technique #32, but the present technique involves a focus on solving specific obstacles to performance.
Instructions on what to do when no specific problems have yet arisen would count as an instance of technique #1 "Provide general information".

22. Set graded tasks, goal setting
Movement towards complex/difficult goals is broken down in simple (but still challenging) steps.
__Participation:__ The person determines whether or not the task is too complex and in which steps the task should be broken down to be manageable. No participation would look like instructions.
__NB__
Might follow from technique #26 "Specific goal setting", but key difference lies in planning to perform a sequence of preparatory actions that increases in difficulty over time, OR breaking down a complex task in manageable subtasks- as opposed to simply planning out a sequence of actions in detail.

23. Reattribution training, external attribution of failure
Help person reinterpret (previous) failure in terms of either unstable and/or changeable attributions and previous successes in terms of stable attributions, OR attribute failure to an external but controllable/avoidable factor so that person remains confident to attempt executing the behavior in the future.

__Intention__

24. General intention formation
Involves setting a general behavioral goal for the person, e.g. take all medication on time, or formulating the desired outcomes of the behavior, e.g. undetectable viral load. It may sometimes be difficult to distinguish this technique from technique #1 "Provide general information", e.g. when written "Goals of treatment are explained". If such a-specific information is presented during an "action planning phase" in the intervention, it is an instance of this technique #24. Otherwise, it can be considered as an instance of technique #1.
__Participation:__ Encourage the person to set a general goal or make a behavioral resolution. In case of no participation, solutions formulated with this technique would look like instructions.
__NB__
Distinguished from technique #26 "Specific goal setting" by the general nature of the goal. Technique #24 **does not involve** planning exactly what will be done or when the behavior/action sequence will be performed.
Different from technique #25 that only concerns planning the time of the day for taking the medication, not any intentional expressions like "I intent to take all medication on time".

25. Develop medication intake schedule
Involves development of a schedule (time) of when to take the medication.
Tailored / Participation: Person is actively involved in determining when the medication intake is planned. May also be referred to as "tailored medication plan".
Medication schedule in writing: Schedule is written down for the patient for the person to take home. Can be combined with pictures of medication/time of intake.
__NB__
Different from techniques #26 as this technique #25 concerns basic planning of medication intake while techniques #26 concerns **goals to change or facilitate adherence** on top of, or after, primary medication intake planning has occurred.
Linking medication intake to daily habits or other cues would also be an instance of technique #30.

26. Specific goal setting
Involves planning ***what*** the person will do including, *at least*, a definition of the goal-directed behaviors that should result in **improved adherence**, **decrease in required efforts to adhere, or undetectable viral load**. This may include the specific contexts in which the behavior will be performed. Without illustration of this level of detail, i.e. formulation of sub-behaviors or preparatory behaviors that should lead to improved levels of adherence, there is not instance of specific goal setting, but rather of General intention formation. The terms "Goal setting", "Personal/action plan" ***do*** suggest that goal-directed behaviors are formulated.
Participation: Encourage the person to develop behavioral goals that fit his/her lifestyle and intentions best. Jointly developed or tailored action plans suggest participation. In case of no participation, solutions formulated look like instructions.
Goals in writing: Goals can be ***written down*** in an action/personal plan for the person to take home.
NB
In case the goal-directed behaviors have been further specified in terms of "**When, where and how or with whom to act**", it is also an instance of implementation intentions in technique #30.
Use of this technique #26 ***does not*** **automatically** imply that technique #24 "General intention formation" has been used.
Different from technique #25 as this technique relates to action plans to increase or facilitate adherence on top of, or after, basic medication intake planning has taken place.

27. Review of general and/or specific goals
Involves reconsideration of previously set goals or intentions following previous goal setting, and an attempt to act on those goals. This technique therefore requires an indication of behavioral performance resulting from Self-monitoring, Self-report or Electronic monitoring of behavior.
Participation: Encourage person to reflect on previously set goals and intentions, and think about whether or not these still suffice. In case of no participation, such reflections are from the professional.
NB
In case **actual behavior** (following period of monitoring) is compared to **desired behavior**, it would be an instance of technique #14 and not of this technique.

28. Agree behavioral contract
Commitment to certain (behavioral) goals formulated in such a manner that non-adherence to these goals would have undesired consequences for the person, e.g. public commitment/signed contract. The person must be aware at the moment of commitment/signing that these intentions will be evaluated in the future.
NB
Different from sub-techniques #25-26 "Medication schedule/goals in writing" because these techniques do not need to involve public commitment or explicit signing. Thus, written personal/action plans would be an instance of technique #26 and not of this technique.

29. Use of social support
Involves prompting the person to think about how others could change their behavior to offer help and/or provide (instrumental) social support and/or provide emotional support. This could also take the form of providing support system as part of the intervention/care (e.g. a "buddy" system).
NB
This could (but does not need to) involve technique #21 "Plan coping responses"- where behavior of people in the environment is perceived to be a barrier to successful performance.

Action control

30. Use of cues
Teach or stimulate person to identify environmental prompts which can be used to remind of the behavior. This could include times of the day, alarm devices, stickers, doses of medication at visible location, particular contexts or elements of contexts, but can also take the form of **Implementation Intentions** by formulating specific goals in terms of "Where, when and how or with whom to act" (requires mental imagery).
NB
Without clear illustration of the level of detail described for implementation intentions, instances are regarded as applications of technique #26 "Specific goal setting". Thus, terms like "Goal setting", "Personal or Action plan" are not enough to ensure inclusion of this technique.
When implementation intentions are used, it automatically implies use of technique #26 "Specific goal setting" but not technique #24 "General intention formation".
When people in the direct environment are asked to help remember intake of medication, the technique to be scored would be #29 "Prompt use of social support".
Cues other than implementation intentions can be used independently or in conjunction with technique #26 "Specific goal setting".

31. Self-persuasion
Encourage the person to use self-motivating strategies to increase motivation and confidence **during periods of behavioral action**. This often takes the form of self-talk, i.e. prompt the person to talk to themselves (aloud or silently) before and during planned behaviors to encourage and support action.

Maintenance

32. Formulate goals for maintenance of behavior
Includes at least method described in #26 "Specific goal setting", may involve techniques #24 "General intention formation", but both focused on maintenance of behavior after change occurred.
Participation: encourage person to develop behavioral goals that fit his/her lifestyle and intentions best. In case of no participation, solutions formulated with this technique would look like instructions.

33. Relapse prevention
Following behavioral change, apply the same method as used in technique #21 "Plan coping responses" but now applied to (long-term) maintenance of behavior.
Participation: prompt *the person* to both determine barriers **and** ways to overcome these. In case of no participation, solutions formulated with this technique would look like instructions.

Facilitation of behavior

34. Provide materials to facilitate behavior, or provide facilities to perform the behavior
Supportive materials are provided to the participants (e.g. reminder devices; dosette box; SMART-cap). Function of the material must be directly related to improvement of the target or preparatory behaviors, not for study-related purposes.
NB
In case the material exists of written goals/instructions/medication schedule, it would be an instance of the sub-techniques "..X... in writing" #25-26.
In case the intervention itself delivered with different materials (e.g. leaflet with information, video, workbook), it is **not** an instance of this technique. Instead, note the use of different intervention materials at the bottom of the table.

35. Continuous professional support
Involves sending letters, making telephone calls, and opportunities for unplanned visits or follow up meetings after the major part of the behavior change intervention has been completed. If these contacts are an intrinsic part of the behavior change intervention, these in themselves do not count as an instance of this technique. Includes the possibility for persons to contact their physician, nurse or other intervention professional in case of any problems.
NB
In case the contacts are intended to serve as cues for behavior or as reminders of formulated goals, it would be an instance of technique #30 and not of this technique.
In case support relates to side-effects, it would be an instance of technique #37.
In case people have the possibility to drop in for unplanned visits during the "behavior change-part" of the intervention, it would be an instance of macro-technique "Tailoring of number of visits".

36. Individualize regimen
In case it is **explicitly mentioned** that regimen type (number of doses, number of pills per doses) is tailored to the needs of the patient, it would be an instance of this technique.
NB
Different from technique #25 "tailored medication plan" which involves tailoring when medication is taken, but not of the regime itself.

37. Cope with side-effects
Physician informs for side-effects and takes steps to deal with these like switching medicines, or providing additional medication to suppress side-effects. Includes descriptions of prompts to contact healthcare professional between visits in case side-effects are experienced.
NB
When people can contact healthcare professional at any time to deal with side-effects, it would be an instance of this technique and not of technique #35.

38. Reduce environmental barriers
Activities aimed at reducing/solving problems that compete for attention with the target behavior, e.g. dealing with unemployment, legal issues, lack of food and housing, etc.

Determinant	#	Technique	1st session: # coded section		Techniques during follow-up. # of sessions:............	
Knowledge	1	Provide general information				
		Tailoring				
		Individualization				
	2	Increase memory & understand.				
Awareness	3	Risk communication				
		Tailoring				
	4	Self-monitoring of behavior				
	5	Self-report of behavior				
	6	Electronic monitoring of behavior				
	7	Reflective listening				
	8	Delayed feedback of behavior				
		Objectively measured [#]				
		Subjectively measured [#]				
	9	Direct feedback of behavior				
	10	Feedback of clinical outcomes				
Social influence	11	Provide info peer behavior				
	12	Social comparison peers				
	13	Norm important other				
Attitude	14	Reevaluation, self-evaluation				
	15	Persuasive communication				
		Tailoring				
	16	Reward behavioral progress				
	17	Reward motivational progress				
Self-efficacy	18	Modeling				
	19	Verbal persuasion				
		Tailoring				
	20	Practice, guided practice				
	21	Plan coping response				
		Participation				
	22	Graded tasks, goal setting				
		Participation				
	23	Reattribution, external attribution				
Intention	24	General intention formation				
		Participation				
	25	Develop medication schedule				
		Tailoring				
		In writing				
	26	Specific goal setting				
		Participation				
		In writing				
	27	Review general or specific goals				
		Participation				
	28	Agree behavioral contract				
	29	Use of social support				
Action control	30	Use of cues				
	31	Self persuasion				
Maintenance	32	Goals for maintenance				
		Participation				
	33	Relapse prevention				
		Participation				
Facilitation	34	Provide materials				
	35	Continuous professional support				
	36	Individualize regimen				
	37	Cope with side effects				
	38	Reduce environmental barriers				

Tailoring of complete intervention strategy? Yes / No | If yes, how?.. | Tailoring number of visits? Yes / No

Format used: Group / Telephone / 1-on-1 counseling / Other................................ If 1-1 or group counseling, who was counselor? ....

Counselor was: 1. Well-trained 3. Received almost no training
2. Received some training 4. Do not know

Additional materials used in the intervention (e.g. brochure):
