# Supplementary material for: A systematic review of hand hygiene improvement strategies: a behavioural approach
Source: Implement Sci. 2012 Sep 14;7:92. doi: 10.1186/1748-5908-7-92 (PMC3517511; doi:10.1186/1748-5908-7-92)
Supplement: Additional file 3 — Worked example data extraction. [file 1748-5908-7-92-S3.pdf]

Worked example of data extraction and coding improvement activities

**Reviewers**	
Name reviewer	TvA
Date	30-03-2010

**Study**	
ID study	55
Title	Controlled trial measuring the effect of a feedback intervention on hand hygiene compliance in a step-down unit
Authors	Blinded
Source + year	2008
Analysed by Naikoba and Hayward	No

**Interventions**
**Description of the intervention**<br><br>**Feedback was provided by the SDU nurse manager who explained the goals and targets for the process measures in the intervention unit twice per week. Feedback was presented to each HCW separately, showing the total number of times the dispensers were used in each patient room in which the HCW worked. These numbers were placed in the medical chart so that the HCWs could review them and improve their performance. They could also review the number of times that other HCWs used dispensers, thus promoting a comparison of hand hygiene compliance among them**
**Description of usual care**<br><br>☐ **Not specified**

Behaviour change techniques used in interventions on hand hygiene compliance			
**Determinant**	**#**	**Method**	**Description of the activity in article**
**Knowledge**	1	☐ Provide general information	
	2	☐ Increase memory and/or understanding of transferred information	
**Awareness**	3	☐ Risk Communications	
	4	☐ Self-monitoring of behaviour	
	5	☐ Self-report of behaviour	
	6	☐ Electronic monitoring of behaviour	
	7	☐ Reflective listening: direct feedback of cognitions and emotions	
	8	☐ **Feedback: delayed feedback of behaviour**	Feedback was presented to each HCW separately, showing the total number of times the dispensers were used in each patient room in which the HCW worked
	9	☐ Feedback: direct feedback of behaviour	
	10	☐ **Feedback of clinical outcomes**	Feedback on infection rates
**Social influence**	11	☐ Provide information about peer behaviour	
	12	☐ **Provide opportunities for social comparison**	review the number of times that other HCWs used dispensers, thus promoting a comparison of hand hygiene compliance among them
	13	☐ Mobilize social norm (important others)	
**Attitude**	14	☐ Re-evaluation of outcomes, self-evaluation	
	15	☐ Persuasive communication, belief selection	
	16	☐ Reinforcement on behavioural progress, provide contingent rewards	
	17	☐ Reinforcement on motivational progress, provide contingent rewards, affirmation	
**Self-efficacy**	18	☐ Modeling	
	19	☐ Verbal persuasion	
	20	☐ Practice, guided practice	

	21	□	Plan coping responses	
	22	□	**Set graded tasks, goal setting**	who explained the goals and targets for the process measures in the intervention unit twice per week.
	23	□	Reattribution training, external attribution	
**Intention**	24	□	**General intention information**	who explained the goals and targets for the process measures in the intervention unit twice per week.
	25	□	Develop medication intake schedule	
	26	□	Specific goal setting	
	27	□	Review of general and/or specific goals	
	28	□	Agree behavioural contract	
	29	□	Use of social support	
**Action control**	30	□	Use of cues	
	31	□	Self-persuasion	
**Maintenance**	32	□	Formulate goals for maintenance of behaviour	
	33	□	Relapse prevention	
**Facilitation of behaviour**	34	□	Provide material to facilitate behaviour	
	35	□	Continuous professional support	
	36	□	Individualize regimen	
	37	□	Cope with side-effects	
	38	□	Reduce environmental barriers	

Reviewers	
Name reviewer	MH
Date	28-02-2010

Study	
ID study	48
Title	Changing knowledge, behavior, and practice related to universal precautions among hospital nurses in China
Authors	Blinded
Source + year	2002
Analysed by Naikoba and Hayward	No

Interventions
**Description of the intervention**<br><br>**Educational training program involving 2 h of formal teaching about blood-borne pathogens and universal precautions delivered by specially trained nurses. One hour demonstration of universal precautions techniques. 30 minute discussion on clarifying risks for blood pathogen exposure 30 min of discussion, and providing printed materials and pamphlets**
**Description of usual care**<br><br>☐ **Not specified**

Behaviour change techniques used in interventions on hand hygiene compliance

Determinant	#	Method	Description of the activity in article
**Knowledge**	1	☐ **Provide general information**	Educational training program involving 2 h of formal teaching about blood-borne pathogens and universal precautions<br>Providing printed materials and pamphlets
	2	☐ Increase memory and/or understanding of transferred information	
**Awareness**	3	☐ **Risk Communications**	30 minute discussion on clarifying risks for blood pathogen exposure
	4	☐ Self-monitoring of behaviour	
	5	☐ Self-report of behaviour	
	6	☐ Electronic monitoring of behaviour	
	7	☐ Reflective listening: direct feedback of cognitions and emotions	
	8	☐ Feedback: delayed feedback of behaviour	
	9	☐ Feedback: direct feedback of behaviour	
	10	☐ Feedback of clinical outcomes	
**Social influence**	11	☐ Provide information about peer behaviour	
	12	☐ Provide opportunities for social comparison	
	13	☐ Mobilize social norm (important others)	
**Attitude**	14	☐ Re-evaluation of outcomes, self-evaluation	
	15	☐ Persuasive communication, belief selection	
	16	☐ Reinforcement on behavioural progress, provide contingent rewards	
	17	☐ Reinforcement on motivational progress, provide contingent rewards, affirmation	
**Self-efficacy**	18	☐ **Modeling**	One hour demonstration of universal precautions techniques

	19	☐ Verbal persuasion	
	20	☐ Practice, guided practice	
	21	☐ Plan coping responses	
	22	☐ Set graded tasks, goal setting	
	23	☐ Reattribution training, external attribution	
**Intention**	24	☐ General intention information	
	25	☐ Develop medication intake schedule	
	26	☐ Specific goal setting	
	27	☐ Review of general and/or specific goals	
	28	☐ Agree behavioural contract	
	29	☐ Use of social support	
**Action control**	30	☐ Use of cues	
	31	☐ Self-persuasion	
**Maintenance**	32	☐ Formulate goals for maintenance of behaviour	
	33	☐ Relapse prevention	
**Facilitation of behaviour**	34	☐ Provide material to facilitate behaviour	
	35	☐ Continuous professional support	
	36	☐ Individualize regimen	
	37	☐ Cope with side-effects	
	38	☐ Reduce environmental barriers	
