# Supplementary material for: A systematic review of hand hygiene improvement strategies: a behavioural approach
Source: Implement Sci. 2012 Sep 14;7:92. doi: 10.1186/1748-5908-7-92 (PMC3517511; doi:10.1186/1748-5908-7-92)
Supplement: Additional file 4 — Calculation of relative difference. [file 1748-5908-7-92-S4.pdf]

Draft paper

PRESENTATION OF DATA FROM EPOC STUDIES (RCTs and CBAs)

Notation:

	**Study**	**Control**
Pre	S_{pre}	C_{pre}
Post	S_{post}	C_{post}
Change	S_{change}	C_{change}

(where $S_{change} = S_{post} – S_{pre}$ and $C_{change} = C_{post} – C_{pre}$)

Data to present:

Pretest mean: S_{pre} vs C_{pre}
Posttest mean: S_{post} vs C_{post}
Absolute change (post): $S_{post} – C_{post}$

Relative percentage change (post): $\frac{(Spost - Cpost)}{Cpost} x100$

Absolute change from baseline: S_{change} vs C_{change}
Difference in absolute change from baseline: $S_{change} - C_{change}$

Example:

	**Study**	**Control**
Pre	27%	40%
Post	55%	51%
Change	28%	11%

Pretest mean: 27% vs 40%
Posttest mean: 55% vs 51%

Absolute change (post): 4%
Relative percentage change (post): 7.8%

Absolute change from baseline: 28% vs 11%
Difference in absolute change from baseline: 17%
