# Supplementary material for: A systematic review of hand hygiene improvement strategies: a behavioural approach
Source: Implement Sci. 2012 Sep 14;7:92. doi: 10.1186/1748-5908-7-92 (PMC3517511; doi:10.1186/1748-5908-7-92)
Supplement: Additional file 5 — Characteristics of excluded studies. [file 1748-5908-7-92-S5.pdf]

Additional file 5: Quality assessment

Excluded due to major limitations: *n*=48

Study (Year)	Design		Content	Sample size	Instruments used	Test statistics	Significance	Level of evidence			Inclusion
	type	ranking						low	moderate	high	
Abbot (2006)	UBA	0	1	0	0	1	1		3*		No
Barreau (2003)	UBA	0	1	0	1	1	1		3*		No
Baker (1998)	UBA	0	1	0	1	0	0	2			No
Berhe (2006)	UBA	0	1	0	0	1	1		3*		No
Bhojani (2008)	UBA	0	0	0	1	0	0	1			No
Bischoff (2000)	UBA	0	1	0	0	1	1		3*		No
Bittner (2002)	UBA	0	1	0	0	1	1		3*		No
Coignard (1998)	UBA	0	1	0	0	1	1		3*		No
Colombo (2002)	UBA	1	1	0	0	0	0	2			No
Connoly (1998)	UBA	0	0	0	0	0	0	0			No
Conrad (2001)	UBA	0	0	0	0	1	1	2			No
Coopersmith (2004)	UBA	0	1	0	0	1	1		3*		No
Cromer (2007)	UBA	0	0	0	1	0	0	1			No
Danchaivijitr (2005-1)	UBA	0	1	0	0	1	1		3*		No
Danchaivijitr (2005-2)	UBA	0	0	0	0	1	1	2			No
Das-Neves (2006)	UBA	0	1	0	0	0	0	1			No
Donowitz (1986)	UBA	0	1	0	1	0	0	2			No
Dubbert (1990)	UBA	0	1	0	0	0	0	1			No
Ebnother (2007)	UBA	0	1	0	0	0	0	1			No
Gill (2009)	UBA	0	1	0	0	1	1	3-			No

Girard (2001)	UBA	0	1	0	0	1	1		3*	No
Graham (1990)	UBA	0	1	0	0	1	1		3*	No
Grayson (2008)	UBA	0	1	0	0	0	1	2		No
Higuera (2005)	UBA	0	1	0	0	1	1		3*	No
Johnson (2005)	UBA	0	1	0	0	1	1		3*	No
Lankfort (2003)	UBA	0	1	0	0	1	1		3*	No
Lederer (2009)	ITS	1	1	0	0	0	0	2		No
Maury (2000)	UBA	0	1	0	0	1	1		3*	No
McGuckin (1999)	UBA	0	1	0	0	1	1		3*	No
McGuckin (2001)	UBA	0	1	0	0	1	1		3*	No
McGuckin (2004)	UBA	0	1	0	0	1	1		3*	No
McGuckin (2004)	UBA	0	1	0	0	0	1	2		No
Ozyazicioglu (2008)	UBA	0	0	0	0	1	1	2		No
Panhotra (2004)	UBA	0	1	0	0	0	1		3*	No
Pessoa-Silva (2007)	UBA	0	1	0	0	1	1	3-		No
Rosenthal (2004)	UBA	0	1	0	0	1	1		3*	No
Rosenthal (2005)	UBA	0	1	0	0	1	1		3*	No
Salemi (2002)	UBA	0	1	0	1	0	0	2		No
Sanchez (2007)	UBA	0	1	0	0	0	1		3-	No
Shaw (2003)	UBA	0	1	0	0	0	0	1		No
Sud (2007)	UBA	0	1	0	0	0	0	1		No
Swoboda (2004)	UBA	0	1	0	0	1	1		3*	No
Swoboda (2007)	UBA	0	1	0	0	1	1		3-	No
Thomas (2005)	UBA	0	1	0	0	0	0	1		No
Tibbals (1996)	UBA	0	1	0	1	0	0	2		No
Tolentino (2007)	UBA	0	1	0	0	0	1	2		No

Venkatesh (2008)	UBA	0	1	0	0	1	1		3-	No
Whitby (2008)	UBA	0	1	0	0	1	1		3-	No
