# Supplementary material for: A systematic review of hand hygiene improvement strategies: a behavioural approach
Source: Implement Sci. 2012 Sep 14;7:92. doi: 10.1186/1748-5908-7-92 (PMC3517511; doi:10.1186/1748-5908-7-92)
Supplement: Additional file 6 — Overview of strategies and methods in the 41 studies reviewed. [file 1748-5908-7-92-S6.pdf]

Additional file 6: Overview of determinants addressed, study methods, and description of quality assessment in the 41 studies reviewed

Study (Year)no.	Strategy<br>Determinants addressed	Methods<br>Design	Setting (n)	Target population	Unit of analysis(n)$	Quality assessment<br>Ranking and limitations
Berg (1995)[33]	Knowledge, Awareness, Self-efficacy, Action control	UBA	Medical/surgical ICU (1)	NS	HH Opportunities (677)<br>Before (251)<br>After (426)	4: Moderate<br>Uncontrolled design, rater procedure not described, test statistics not described
Brock (2002)[34]	Awareness	RCT	Study: Emergency care (1)<br>Control: ICU (1)	Nurses<br>Technical staff	Participants (90)<br>Study (45 nurses)<br>Control (45 nurses)	5: Moderate<br>Obtrusive observations, rater procedure not described
Brown (2003)[35]	Knowledge, Awareness, Social influence, Self-efficacy, Intention, Action control, Facilities	UBA	NICU (1)	Nurses<br>Physicians<br>Other HCWs	HH Opportunities (606)<br>Before (283)<br>After (323)	4: Moderate<br>Uncontrolled design, sample size described but not justified, rater procedure not described
Conly (1989)[36]	Knowledge, Awareness, Social influence, Attitude, Action control	UBA	Medical ICU (1)	Nurses<br>Physicians<br>Other HCWs	HH Opportunities (211)<br>Before (122)<br>After period (99)	4: Moderate<br>Uncontrolled design, sample size described but not justified, rater procedure not described
Creedon (2006)[37]	Knowledge, Awareness, Action control, Facilities	UBA	Medical/surgical ICU (1)	Nurses<br>Physicians<br>Other HCWs	HH Opportunities (314)<br>Before (152)<br>After (162)	4: Moderate<br>Uncontrolled design, obtrusive observations, rater procedure not described
Dorsey (1996)[38]	Knowledge, Action control	UBA	Emergency department (1)	Nurses<br>Physicians<br>Other HCWs	HH Opportunities (252)<br>Before (132)<br>After (120)	4: Moderate<br>Uncontrolled design, sample size described but not justified/rater procedure not described
Duerink (2006)[39]	Knowledge, Awareness, Self-efficacy, Action control, Facilities	CBA	Study: Medical internal ward (1)<br>Paediatric ward (1)<br>Control: Gynaecology ward (1)	NS	HH Opportunities (7160)<br>Study (5005)<br>Control (2155)	5: Moderate<br>sample size not described, rater procedure not described
Earl (2001)[40]	Facilitation of behaviour	UBA	Medical ICU (1)<br>Surgical ICU (1)	Nurses<br>Physicians<br>Other HCWs	HH Opportunities (2181)<br>Before (1090)<br>After (1091)	4: Moderate<br>Uncontrolled design, sample size described but not justified, rater procedure not described
Eldridge (2006)[41]	Knowledge, Awareness, Social influence, Action control, Facilities	UBA	3 Medical centres ICU (4)	Nurses<br>Physicians<br>Other HCWs	HH Opportunities (4103)<br>Before (2338)<br>After (1762)	4: Moderate<br>Uncontrolled design, obtrusive observations, rater procedure not

						described
Giannitsioti (2009)[42]	Facilitation of behaviour	UBA	Medical ward (1)	Nurses<br>Physicians<br>Other HCWs	HH Opportunities (299)<br>Before (207)<br>After (92)	4: Moderate<br>Uncontrolled design, sample size described but not justified, rater procedure not described
Golan (2006)[43]	Action control	Cross-over	Medical ICU (1)<br>Surgical ICU (1)	Nurses<br>Physicians<br>Other HCWs	HH Opportunities (1619)<br>Medical ICU (998)<br>Surgical ICU (621)	6: high<br>rater procedure not described
Gould (1997)[44]	Knowledge, Awareness, Attitude, Self-efficacy, Facilities	CBA	Study:<br>Surgical wards (2)<br>Control:<br>Surgical wards (2)	Nurses	Participants<br>Study (16)<br>Control (15)	4: Moderate<br>Sample size described but not justified, obtrusive observations, rater procedure not described
Haas (2008)[45]	Facilitation of behaviour	UBA	Emergency department (1)	Nurses<br>Physicians<br>Other HCWs	HH Opportunities (757)<br>Before NS<br>After NS	4: Moderate<br>Uncontrolled design, obtrusive observations, rater procedure not described
Harbarth (2002)[46]	Knowledge, Awareness, Social influence, Attitude, Action control, Facilities	UBA	General PICU (1),<br>Cardiac ICU (1),<br>NICU (1)	Nurses<br>Physicians<br>Other HCWs	HH Opportunities (12,216)	5: Moderate<br>Uncontrolled design, sample size described but not justified
Howard (2009)[47]	Knowledge, Awareness, Action control	UBA	Surgical ward (4)<br>Emergency department (1)	Nurses<br>Physicians<br>Other HCWs	Patients<br>Before (85 patients)<br>After (74 patients)	4: Moderate<br>Uncontrolled design, sample size described but not justified, rater procedure not described
Huang (2002)[48]	Knowledge, Awareness, Self-efficacy	RCT	All hospital departments	Nurses	Participants (98)<br>Study (49 nurses)<br>Control (49 nurses)	4:Moderate<br>Sample size described but not justified, obtrusive observations, rater procedure not described
Khatib (1999)[49]	Action control	UBA	ICU (1)	NS	HH Opportunities (1080)<br>Before (537)<br>After (543)	3: Moderate<br>Uncontrolled design, sample size described but not justified, rater procedure not described, test statistics not described
Lam (2004)[50]	Knowledge, Attitude, Self-efficacy, Action control, Facilities	UBA	NICU (1)	Nurses<br>Physicians<br>Other HCWs	HH Opportunities (983)<br>Before (666)<br>After (317)	4: Moderate<br>Uncontrolled design, sample size described but not justified, rater procedure not described
Larson (1991)[51]	Knowledge, Facilities	UBA	NICU*(1)<br>Recovery unit (1)	Nurses<br>Physicians<br>Other HCWs	HH Opportunities (1610)<br>Automatic sink (615)<br>Manual sink (995)	4: Moderate<br>Uncontrolled design, sample size described but not justified, obtrusive

						observations but rater procedure described
Larson (1997)[52]	Knowledge, Awareness, Social influence, Facilities	CBA	Study:<br>Neurosurgical ICU (1)<br>Control:<br>Surgical ICU (1)	Nurses	HH Opportunities (1983)<br>Study (991)<br>Control (992)	6: High<br>Sample size described but not justified
Larson (2000)[53]	Knowledge, Awareness, Social influence, Attitude, Self-efficacy, Intention, Action control	CBA	Study: 1 hospital<br>Medical ICU (1)<br>NICU (1)<br>Control: 1 hospital<br>Medical ICU (1)<br>NICU (1)	NS	Number of dispenser activations (860,567)<br>Study (477,680)<br>Control (382,887)	4: Moderate<br>Sample size described but not justified, adherence measured by used volume of soap or hand alcohol
Larson (2005)[54]	Facilitation of behaviour	Cross-over	Paediatric emergency department (1)<br>PICU (1)	NS	HH Opportunities (5568)	6: High<br>Obtrusive observations but rater procedure described
Marra (2008)[55]	Awareness, Social influence, Self-efficacy, Intention	CBA	Study: step-down unit (1)<br>Control: step-down unit (1)	HCWs (NS)	HH episodes by electronic counting device (228,297)<br>study (117,579)<br>Control (110,718)	4: Moderate<br>Sample size described but not justified, adherence measured by electronic counting device
Mayer (1986)[56]	Knowledge, Awareness, Facilities	CBA	Study: ICU (1)<br>Control: ICU (1)	Nurses	HH opportunities (834)<br>Study (610)<br>Control (224)	6: High<br>Sample size described but not justified
Moongtui (2000)[57]	Awareness, Social influence	RCT	Emergency department (1)<br>Randomisation of nurses	Nurses<br>Other HCWs	Participants (91)<br>Study (36)<br>Control (55)	6: High<br>Rater procedure not described
Muto (2000)[58]	Knowledge, Self-efficacy, Action control, Facilities	UBA	Medical ward (1)<br>Medical ICU (1)	Nurses<br>Physicians<br>Other HCWs	HH Opportunities (239)<br>Before (126)<br>After (113)	3: Moderate<br>Uncontrolled design, rater procedure not described, test statistics not described
Picheansathian (2008)[59]	Knowledge, Awareness, Action control, Facilities	UBA	PICU (1)	Nurses	HH Opportunities (1245)<br>Before (320)<br>After (925)	4: Moderate<br>Uncontrolled design, sample size described but not justified, obtrusive observations, rater procedure described
Pittet (2000)[1]	Knowledge, Awareness, Social influence, Action control, Facilities	UBA	All hospital wards	Nurses<br>Physicians<br>Other HCWs	HH Opportunities (5403)<br>Before (2834<br>After (2569)	5: Moderate<br>Uncontrolled design, sample size described but not justified
Raju (1991)[60]	Knowledge, Awareness, Attitude	UBA	NICU (1)	Nurses<br>Physicians	HH Opportunities (412)<br>Before (257)	4: Moderate<br>Uncontrolled design, sample size

				Other HCWs	After (155)	described but not justified, rater procedure not described
Raskind (2007)[61]	Knowledge, Intention, Action control	UBA	PICU (1)	Nurses<br>Physicians<br>Other HCWs<br>Administrative personnel,<br>Family and visitors	HH Opportunities (401)<br>Before (189)<br>After (212)	4: Moderate<br>Uncontrolled design, sample size described but not justified, rater procedure not described
Rupp (2007)[62]	Knowledge, Action control, Facilities	Cross-over	ICU A/B	Nurses<br>physicians<br>other HCWs	HH Opportunities (3678)	5: Moderate<br>Sample size described but not justified, rater procedure not described
Santana (2007)[63]	Knowledge, Action control, Facilities	UBA	ICU (1)	Nurses<br>Physicians<br>Other HCWs	HH Opportunities (3476)<br>Before (2032)<br>After (1444)	4: Moderate<br>Uncontrolled design, sample size described but not justified, rater procedure not described
Sharek (2002)[64]	Knowledge, Awareness, Action control	UBA	NICU (1)	Nurses<br>Physicians<br>Other HCWs	Participants (46)	4: Moderate<br>Uncontrolled design, sample size described but not justified, rater procedure not described
Simmons (1990)[65]	Knowledge, Awareness, Social influence, Attitude, Action control	UBA	Surgical ICU (1)<br>Medical ICU (1)	Nurses<br>Physicians<br>Other HCWs	HH Opportunities (485)<br>Before (257)<br>After (155)	4: Moderate<br>Uncontrolled design, sample size described but not justified, rater procedure not described
Slota (2001)[66]	Knowledge, Action control	UBA	PICU (1)	NS	HH Opportunities (350)<br>Before and after numbers NS	4: Moderate<br>Uncontrolled design, sample size described but not justified, rater procedure not described
Trick (2006)[67]	Knowledge, Awareness, Attitude, Action control, Facilities	CBA	Multicentre (4)<br>study: 3 hospitals<br>Medical ward (2)<br>Surgical ward (1)<br>ICU/PICU (5)<br>Skilled care (1)<br>Control: 3 hospitals<br>ICU/PICU (2)<br>Rehabilitation ward (1)	Nurses<br>Physicians<br>Other HCWs	HH Opportunities (6948)<br>Study (5206)<br>Control (1742)	6: High<br>Sample size described but not justified
v/d Mortel	Awareness, Action control	UBA	NICU (1)	Nurses	HH Opportunities (893)	4: Moderate

(1995)[68]			High care (1)	Physicians<br>Other HCWs	Before (303)<br>After (590)	Uncontrolled design, sample size described but not justified, rater procedure not described
v/d Mortel (2000)[69]	Awareness	UBA	ICU (1)<br>High care (1)	Nurses<br>Physicians<br>Other HCWs	HH Opportunities (542)<br>Before (143)<br>After (399)	4: Moderate<br>Uncontrolled design, sample size described but not justified, rater procedure not described
Whitby (2004)[70]	Facilitation of behaviour	UBA	Internal medical ward (1)<br>PICU (1) Infectious diseases ward (1)<br>Urology ward (1)	Nurses	HH Opportunities (8146)<br>Before (4001)<br>After (4145)	4: Moderate<br>Uncontrolled design, sample size described but not justified, rater procedure not described
Won (2004)[71]	Knowledge, Awareness, Attitude, Action control, Facilities	UBA	NICU (1)	Nurses<br>Physicians<br>Other HCWs	HH Opportunities NS (312 observation periods)<br>Before NS<br>After NS	4: Moderate<br>Uncontrolled design, sample size not described, rater procedure not described
Zerr (2005)[72]	Knowledge, Awareness, Social influence, Attitude, Self-efficacy, Action control, Facilities	UBA	Paediatric ward (1)<br>Surgical ward (1)	Nurses<br>Physicians<br>Other HCWs	HH Opportunities (1526)<br>Before (958)<br>After (568)	4: Moderate<br>Uncontrolled design, sample size described but not justified, rater procedure not described

CBA = Controlled before-and-after study, HH = hand hygiene, HCWs = healthcare workers, ICU = intensive care unit, NICU = neonatal intensive care unit, NS = , PICU = paediatric intensive care unit, UBA = uncontrolled before-and-after study,

$Numbers are calculated from baseline data and data derived directly
