# Supplementary material for: A systematic review of hand hygiene improvement strategies: a behavioural approach
Source: Implement Sci. 2012 Sep 14;7:92. doi: 10.1186/1748-5908-7-92 (PMC3517511; doi:10.1186/1748-5908-7-92)
Supplement: Additional file 7 — Details of study characteristics, improvement activities and results in the 41 studies reviewed. [file 1748-5908-7-92-S7.pdf]

Additional file 7: Details of study characteristics, improvement activities and results in the 41 studies reviewed

**Study (Year)[no.]**	**Region**	**Theory based[*]**	**Analysis of barriers[†]**	**Description of improvement activities**	**Results on HH compliance[‡]**
Berg (1995)[33]	Central America			A nontargeted intervention in conjunction with ongoing structured education. Placing signs at patient's bedside in case of a present pathogen. Lectures, demonstration of HH technique, positive and negative feedback. Managers of the wards encouraged HH practice.	Before: 5% (13/251)<br>After: 63% (268/426)<br>Absolute difference: 58%
Brock (2002)[34]	USA	Social cognitive theory	Yes	Performance feedback was given to the intervention group on a personal confidential card, which identified the individual's rate of HW and HW technique.	After intervention vs control:<br>80·1 (47 nurses) - 68·1 (45 nurses) = 12/68·1*100<br>Relative difference: 17·6
Brown (2003)[35]	Russia			Personalised instruction from infection control nurse, more personalised instruction from a role model. Identification and training of opinion leader (spoke with individual staff members). Display of colonisation rates. Corrected HCWs with poor HH and nosocomial infections. Demonstration of colony forming on fingers. Alcohol-based hand rub provided. Dispensers at each bed. Working group formed. All nursing staff required to sign a statement outlining requirements for HH.	Before: 44% (125/283)<br>After: 48% (155/323)<br>Absolute difference: 4%
Conly (1989)[36]	Canada			Policies and procedures reviewed and modified. Infection control staff emphasised importance of HH. Deficiencies emphasised in service rounds. Feedback data about poor practice. Results of previous surveys presented. Memoranda sent to staff and department. Posters emphasising procedures placed in the MICU.	Before: 21% (26/122)<br>After: 49% (44/89)<br>Absolute difference: 28%
Creedon (2006)[37]	Europe	Precede model		A multifaceted HH programme: provision of knowledge (an educational hand-out and poster campaign), enabled by provision of an alcohol rub; and HH behaviour was reinforced by feedback of baseline observations on posters. Rationale of HH, Nosocomial infection rates and costs, HH technique, knowledge transmitters and behavioural prompts. Feedback on results of HH behaviour (baseline) in poster format. New alcohol hand rub.	Before: 51% (78/152)<br>After: 83% (134/162)<br>Absolute difference: 32%
Dorsey (1996)[38]	USA			Distribution of a HW-related publication to all staff. Brightly coloured fluorescent signs with CDC HW recommendations were posted al all sinks.	Before: 52% (69/133)<br>After: 61% (71/116)<br>Absolute difference: 9%
Duerink (2006)[39]	Asia			The intervention consisted of the development of a protocol for standard precautions, installation of washstands, educational activities and performance feedback. Summary of a protocol for each attendant. Brightly coloured posters depicting the procedures in nurses' rooms.	After intervention vs control:<br>90 (unclear) - 17 (unclear) = 73/17*100

				Lecture on standard precaution. Practical sessions in small groups. Charts and oral feedback. Distribution of pocket calculator with statements on infection control as small gift. Installation of washstands. Individual bottles of hand alcohol. Hand alcohol in all rooms of the ward.	Relative difference: 429
Earl (2001)[40]	USA			Introduction of an new alcohol-based hand rub.	Before: 39·6% (432/1090)<br>After: 52·6% (574/1091)<br>Absolute difference: 13%
Eldridge (2006)[41]	USA	Six sigma model	Yes	Alcohol-based hand rubs for each bed, pocket-sized alcohol hand rubs, hand lotion, soap. Posters, brochure or sign to promote alcohol-based hand rubs. One-page summary document of CDC guidelines. An 8-min video about HH. Annual compliance measurement. Posters and buttons for patients and visitors: 'It's OK to ask health care providers if they cleaned their hands.'	Before: 47% (1099/2338)<br>After: 80% (1410/1762)<br>Absolute difference: 33%
Giannitsioti (2009)[42]	Europe			Installation of a alcohol-based hand rub antiseptic bed rail system.	Before: 36% (207)<br>After: 36% (92)<br>Absolute difference: 0%
Golan (2006)[43]	USA			The intervention consisted of eliminating the gown-use requirement from the contact precautions protocol for patients infected or colonised with vancomycin-resistant enterococcus or methicillin-resistant *Staphylococcus aureus*.	After: intervention vs control:<br>1619 observations in total<br>37 (unclear) - 34 (unclear) = -3<br>Relative difference: -8·8
Gould (1997)[44]	Europe			Educational sessions included theory and practical demonstrations of HW. Information about and importance of transmission and decontamination. Demonstration of ideal technique. Risk in relation to blood and body fluid contact. Reinforcement of technique and practice with feedback.	After: intervention vs control<br>58·6 (16 nurses) - 64·1 (15 nurses) = -5·5/64·1*100<br>Relative difference: -8·6
Haas (2008)[45]	USA		Yes	Introduction of hand gel to personnel.	Before: 43% (total 757)<br>After: 51%<br>Difference: 8%
Harbarth (2002)[46]	USA			Introduction of an alcohol-based hand gel; multifaceted quality improvement interventions (educational programme, opinion leaders, performance feedback).Handout toolkit with educational materials and key journal papers for opinion leaders, educational sessions about the importance of HH and misconceptions about alcohol-based HH, personal reminders from opinion leaders. Performance feedback of HH	12,216 observations in total<br>Before: 28·2% (unclear)<br>After: 37·2% (unclear)<br>Absolute difference: 9%

			compliance data in graphic form. Double page-size coloured posters. Multidisciplinary meetings.	
Howard (2009)[47]	Europe		Distribution of a 'clean practice protocol' poster to raise awareness of key infection-control activities, as advised by the latest international guidelines. Clean practice protocol poster used for education at the multidisciplinary team meetings and on surgical wards to remind staff of clean practice requirements. Results of audits were presented to staff.	Before: 28% (85)<br>After: 87% (74)<br>Absolute difference: 59%
Huang (2002)[48]	Asia		Educational training programme involving 2 h of formal teaching about blood-borne pathogens and universal precautions delivered by specially trained nurses, 1 h of practical demonstration, 30 min of discussion, and written information.	After intervention vs control:<br>88·8 (49 nurses) 62·4 (49 nurses)= 26·4/62·4*100<br>Relative difference: 42·3
Khatib (1999)[49]	Asia		Permanently placed warning labels on mechanical ventilators to remind staff of HW and the use of surgical gloves by respiratory care practitioners in the ICU.	Before: 64·5% (346/537)<br>After: 92% (500/543)<br>Absolute difference: 27·5%
Lam (2004)[50]	Asia		The intervention consisted of problem-based and task-oriented HH education, enhancement of minimal handling protocol and clustering of nursing care, liberal provision of alcohol-based hand antiseptic, improvement in HH facilities. A HH protocol was incorporated as part of the orientation programme for all new staff, emphasising the importance and the correct steps of HW. Step-by-step protocols for common nursing procedures were developed and implemented by face-to-face training and return demonstration that were conducted at regular intervals. Pictures on steps of correct HW procedures were posted at each HW basin. Antiseptic alcohol-based hand rub was made readily available. Water taps of wash basins modified to allow hands-free operation by fitting an infrared automatic sensor.	Before: 39·5% (263/666)<br>After: 56% (178/317)<br>Absolute difference: 16·5%
Larson (1991)[51]	USA		Introduction of an automated sink.	Before: 61·8% (995/1610)<br>After: 38·2% (615/1610)<br>Absolute difference: -23·6%
Larson (1997)[52]	USA	Precede model	A multifaceted intervention including focus group sessions, installation of automated sinks, and feedback to staff on HW frequency. Sessions to reinforce learning. Focus group sessions with staff about HW practices and beliefs. Suggestions for new methods were reviewed. Contradictions were examined, and group process was used to develop a unit-based plan to improve HW. Feedback on HW frequencies (posted bar charts	After intervention vs control:<br>83 (190/229) - 48 (75/157) = 35/48*100<br>Relative difference: 73

			weekly). Installation of automatic sinks; full sequence mode in phase 4. Active and visible involvement and support of the units' administration.	
Larson (2000)[53]	USA		Programme consisted of education, feedback, reaction to outbreaks, HH products for home use, role modelling, management commitment, involvement and support, allocation of rewards. HW fact sheet developed and distributed. Educational programme about HH procedures. Unit-specific feedback data about infection rates weekly. Outbreaks and high infection rates used to review and reinforce HH compliance expectations. Article describing correct procedure published in two hospital publications. Sample of HH products for use at home. All personnel in supervisory role encouraged to role model HH and to point out poor HH in others. Selection of individuals for formal recognition (allocation of rewards. All new ones signed a copy of the HH sheet. Letter from chief executive officer and medical director stating their commitment.	After intervention vs control: (number of soap dispensing/patient care days): 42 (148,562/3458) – 39·2 (132,944/3389) = 3·8/39·2*100 Relative difference: 9·7
Larson (2005)[54]	USA		Introduction of manual and touch-free dispensers of alcohol sanitiser.	After: intervention vs control (number of uses per dispenser per day/ mean) 41·2 - 25·6 = 15·6/26·6*100 Relative difference: 61
Marra (2008)[55]	South America		Feedback was provided by the nurse manager of the step-down units who explained the goals and targets for the process measures in the intervention unit twice per week. Feedback was presented to each HCW separately, showing the total number of times the dispensers were used and promoting a comparison of HH compliance among HCWs.	After intervention vs control (rate of use, number of HH episodes) 41·1 - 35·8 = 5·3/35·8*100 Relative difference: 14·8
Mayer (1986)[56]	USA	Yes	The introduction of a moisturised soap and feedback in the form of daily memos to individual staff about the previous day's HW. Written description of the three behaviour categories en critical procedures that should be followed. Feedback on HW frequency from previous day. Changing HH agent in emollient HW product.	After intervention vs control: 92 (157) - 77 (53) = 15/77*100 Relative difference: 19·5
Moongtui (2000)[57]	Asia		Peer feedback programme. Open observations of HH practice by peers. Posted feedback of compliance at group level every 3 days.	After: intervention vs control 82·7 (36 nurses) - 65·8 (55 nurses) = 16·9/65·8*100 Relative difference: 25·7

Muto (2000)[58]	USA		Introduction of alcohol-based hand antiseptics accompanied with an educational campaign with 4 weekly visits to these floors to remind and reinstruct staff about the use of the alcohol dispensers and to address questions. Meeting with staffs. Placement of signs in common area. Messages inserted in the wards' communication books to remind staff about new dispensers. Placement of signs in common area. Alcohol dispensers were mounted next to every door. Educational and motivational campaign. Three weekly visits to motivate and reinstruct staff and address any questions and comments about HH and dispensers.	Before: 60% (76/126)<br>After: 52% (59/113)<br>Absolute difference: -8%
Picheansathian (2008)[59]	Asia		The intervention was a HH promotion programme including a training session, regular performance feedback and reminder poster displays. Provision of bedside alcohol-based solution, distribution of individual bottles of alcohol-based hand rub.	Before: 6·3% (320)<br>After: 81·2% (925)<br>Absolute difference: 75%
Pittet (2000)[1]	Europe	Yes	A hospital-wide programme with special emphasis on bedside, alcohol-based hand disinfection, reminders (posters), feedback and encouragement from senior staff. Involvement and support of hospital management. Participation in regular meetings of project team. Performance feedback twice a year. Visual display of double-page-size colour posters that emphasise the importance of HH. Individual bottles of alcohol hand rub. Custom-made holders were mounted on all beds. Promotional material (talking walls).	Before: 48% (1360/2834)<br>After: 66% (1696/2569)<br>Absolute difference: 18%
Raju (1991)[60]	USA	Yes	The programme included four interventions, namely, five educational sessions (importance of HH related to nosocomial infections, methods of prevention, rationale for HH prophylaxis), in-service training during ward rounds, distribution of literature about HW and feedback of monitoring results from compliance and bacterial cultures.	Before: 28·4% (73/257)<br>After: 62·6% (97/155)<br>Absolute difference: 34·2%
Raskind (2007)[61]	USA		An educational programme that used a range of educational materials, including illustrations and a written description of proper HH techniques specific to the NICU. The educational materials reinforced the importance, frequency and included illustrations and a written description of proper HH techniques specific to the NICU. These materials were disseminated by means of an e-mailed brochure. Prominently displayed bulletins and posters that described proper required HH in the NICU.6Verbal reminders.	Before: 89% (168/189)<br>After: 100% (212/212)<br>Absolute difference: 11%
Rupp (2007)[42]	USA		An educational programme regarding HH in both units, consisting of face-to-face meetings with nursing staff (and mandatory videotaped viewing for the night shift), a questionnaire with hand-outs, and posting of reminder signs. Dispensers for alcohol-based hand gel.	After intervention vs control:<br>(3768 observations in total)<br>68·5 – 37·5 =

			31/37·5*100<br>Relative difference: 82·7
Santana (2007)[63]	South America	Eight dispensers containing alcohol-based hand gel were introduced (1 dispenser for every 2 beds). Five page-sized colour posters were placed strategically around the unit to emphasise the importance of HH.<br>A sticker was placed on each alcohol dispenser instructions for use and to encourage HH. Ten educational sessions lasting approximately 15 min.	Before: 18·3% (372/2032)<br>After: 20·8% (300/1444)<br>Difference: 2·5%
Simmons (1990)[64]	USA	The interventions included compulsory attendance of in-service training, distribution of educational material, distribution of buttons encouraging HCWs to wash their hands, feedback, and on-the-spot critique of HW for all staff. Delivery of two key publications. In-service rounds by physician about importance of HH and HH indications. Open observations and direct feedback (deficiencies specially pointed out). Control staff wore and handed out buttons to encourage HCWs to proper HH.	Before: 22% (39/177)<br>After: 29·9% (92/308)<br>Absolute difference: 7·9%
Sharek (2002)[65]	USA	An evidence-based HW policy, supported by an intensive education programme, reminders, and feedback. Educational notices and educational sessions. Feedback on compliance data and infection rates. Stickers and posters.	Before: 47·4% (n = 19)<br>After: 85·4% (n = 48)<br>Absolute difference: 38%
Slota (2001)[66]	USA	Strict HW and protective gown and glove use. In-service training. One-to one education with each administered patient to study. Large free-standing signs with direction placed in front of each patient's bed.	Before: 22% (not clear)<br>After: 76% (350)<br>Absolute difference: 54%
Trick (2006)[67]	USA	45-min educational sessions for personnel during their annual mandatory infection control education programmes. Components of the presentation included review of the CDC's Healthcare Infection Control Practices 45-min educational sessions. Data on hospital-specific HH adherence. Pocket-sized bottles of alcohol-based hand rub. An HH fact sheet. Alcohol-based hand rub was readily available in all inpatient care areas. Hospital-wide poster campaigns at the intervention hospitals. The campaign featured humorous posters of high-profile hospital administrative and clinical staff using and encouraging HCWs to use alcohol-based hand rubs.	After: intervention vs control:<br>(6948 observations in total)<br>46·3 - 31 = 15·3/31*100<br>Relative difference: 49·5
v/d Mortel (1995)[68]	Australia	Six weekly feedback in the form of charts of non-personalised HW performance. Charts of revised HW performance were displayed about the sinks at 6 weeks intervals.	Before: 63% (191/303)<br>After: 63% (772/590)<br>Absolute difference: 0%
v/d Mortel (2000)[69]	Australia	Feedback about compliance rates.	Before: 61% (87/143)<br>After: 83% (331/399)<br>Absolute difference: 22%

Whitby (2004)[70]	Australia		Sinks positioned at the bedside as well at the entrance to patients room.	Before: 49% (1960/4001)<br>After: 35% (1451/4145)<br>Absolute difference: -14%
Won (2004)[71]	Asia		The HH campaign. Hibiscrub and Better-Iodine were the antimicrobial soaps used for soap-and-water HW during the study period. A special educational programme with formal lectures about the appropriate use of each hand-cleansing agent. Cartoons showing correct HH technique posted above sinks. Folders of written instructions. Formal lectures (appropriate use of agents, correct HH techniques, importance of HH). Feedback about compliance rates monthly. Errors were privately discussed with individual HCWs. Labels with slogan in visible sites. Financial incentives and penalties. Public praise from head nurse.	Before: 43% (unclear)<br>After: 74% (unclear)<br>Absolute difference: 31%
Zerr (2005)[72]	USA	Social cognitive theory	A hospital-supported, house-wide campaign. Intervention 1 was initiated after period 1 and consisted of education targeting nurses and parents of patients. The goal of intervention 2 was to change HH behaviour through intensive education. Written information for parents, technique, and objective HH. Posters. Temporary tattoos for children. Education about alcohol hand gel. Conferences (hospital grand rounds). Employee newspaper. Dissemination of small bottles of hand alcohol. Placement of hand gel dispensers. Feedback during hospital grand rounds about hospital's own data about HAI and HH over time. Mailings and signs. Written materials invited parents to remind staff to clean their hands. Formulation of multidisciplinary team and use of role models.	Before: 62% (958)<br>After: 74% (568)<br>Absolute difference: 12%

CDC = Centres for Disease Control, HH = hand hygiene, HAI = hospital-acquired infection, HCW = healthcare worker, HW = hand washing, ICU = intensive care unit, MICU = mobile intensive care unit, NICU = neonatal intensive care unit

* Strategy is theory based

[†]Strategy based on analysis of barriers by practice research

[‡]Absolute difference = % After - % Before

Relative difference = 100 * (Intervention – Control)/Control
